# Immune Response of Calves Vaccinated with *Brucella abortus* S19 or RB51 and Revaccinated with RB51

**DOI:** 10.1371/journal.pone.0136696

**Published:** 2015-09-09

**Authors:** Elaine M. S. Dorneles, Graciela K. Lima, Andréa Teixeira-Carvalho, Márcio S. S. Araújo, Olindo A. Martins-Filho, Nammalwar Sriranganathan, Hamzeh Al Qublan, Marcos B. Heinemann, Andrey P. Lage

**Affiliations:** 1 Laboratório de Bacteriologia Aplicada, Departamento de Medicina Veterinária Preventiva, Escola de Veterinária, Universidade Federal de Minas Gerais, Belo Horizonte, Minas Gerais, Brazil; 2 Laboratório de Biomarcadores de Diagnóstico e Monitoração, Centro de Pesquisas René Rachou, Fundação Oswaldo Cruz, Belo Horizonte, Minas Gerais, Brazil; 3 Center for Molecular Medicine and Infectious Diseases, Department of Biomedical Sciences and Pathobiology, Virginia Maryland College of Veterinary Medicine, Virginia Polytechnic Institute and State University, Blacksburg, Virginia, United States of America; 4 Departamento de Medicina Veterinária Preventiva e Saúde Animal, Faculdade de Medicina Veterinária e Zootecnia da Universidade de São Paulo, São Paulo, São Paulo, Brazil; Universidad Nacional de la Plata, ARGENTINA

## Abstract

*Brucella abortus* S19 and RB51 strains have been successfully used to control bovine brucellosis worldwide; however, currently, most of our understanding of the protective immune response induced by vaccination comes from studies in mice. The aim of this study was to characterize and compare the immune responses induced in cattle prime-immunized with *B*. *abortus* S19 or RB51 and revaccinated with RB51. Female calves, aged 4 to 8 months, were vaccinated with either vaccine S19 (0.6–1.2 x 10^11^ CFU) or RB51 (1.3 x 10^10^ CFU) on day 0, and revaccinated with RB51 (1.3 x 10^10^ CFU) on day 365 of the experiment. Characterization of the immune response was performed using serum and peripheral blood mononuclear cells. Blood samples were collected on days 0, 28, 210, 365, 393 and 575 post-immunization. Results showed that S19 and RB51 vaccination induced an immune response characterized by proliferation of CD4^+^ and CD8^+^ T-cells; IFN-ɣ and IL-17A production by CD4^+^ T-cells; cytotoxic CD8^+^ T-cells; IL-6 secretion; CD4^+^ and CD8^+^ memory cells; antibodies of IgG1 class; and expression of the phenotypes of activation in T-cells. However, the immune response stimulated by S19 compared to RB51 showed higher persistency of IFN-ɣ and CD4^+^ memory cells, induction of CD21^+^ memory cells and higher secretion of IL-6. After RB51 revaccination, the immune response was chiefly characterized by increase in IFN-ɣ expression, proliferation of antigen-specific CD4^+^ and CD8^+^ T-cells, cytotoxic CD8^+^ T-cells and decrease of IL-6 production in both groups. Nevertheless, a different polarization of the immune response, CD4^+^- or CD8^+^-dominant, was observed after the booster with RB51 for S19 and RB51 prime-vaccinated animals, respectively. Our results indicate that after prime vaccination both vaccine strains induce a strong and complex Th1 immune response, although after RB51 revaccination the differences between immune profiles induced by prime-vaccination become accentuated.

## Introduction

The genus *Brucella* causes brucellosis, one of the major zoonosis in public and animal health, that affects livestock and wildlife animal species as well as humans [1,2]. Cattle are the preferred host of *Brucella abortus* [1] and the economic importance attributed to bovine brucellosis is based on direct losses caused by abortions, stillbirths, weight loss, decreased milk production and the establishment of sanitary barriers to international trade of animals and their products [3].

Vaccination is the most effective measure to reduce the prevalence of brucellosis and it has contributed enormously to the success of many control programs [4]. Currently, S19 and RB51 are the *B*. *abortus* vaccine strains more widely used to prevent brucellosis in cattle [5]. Both vaccines are effective in the prevention of abortion and infection, besides offering long lasting protection [5–13]. *B*. *abortus* S19 is a stable smooth attenuated organism with high immunogenicity and antigenicity [14]. It has been used to prevent brucellosis for more than seven decades. RB51 vaccine is a lipopolysaccharide O-antigen deficient naturally occurring rough mutant derived from the virulent smooth strain, *B*. *abortus* 2308 [15]. Therefore, RB51 does not induce antibodies against smooth lipopolysaccharide (LPS) detectable by routine serological tests [15]. This feature allows RB51 vaccination to be performed at any age, while vaccination with S19 is normally restricted to calves between 3 and 8 months of age to avoid interference in the routine serological tests results [2,16].

Currently, almost all the knowledge available on the protective response induced by both *B*. *abortus* vaccine strains comes from research using the mouse model [17–20]. Studies in mice have shown that S19 and RB51 induce a strong Th1 cell-mediated immune response with production of IFN-ɣ but not IL-4 in immunized animals, besides CD8^+^ specific cytotoxic T-cells [18,19,21–31]. In contrast, the immune mechanism used by *B*. *abortus* vaccines to confer protection in cattle is unclear. T lymphocyte response induced by *B*. *abortus* vaccination in cattle has been extensively evaluated, but only through proliferation assays [32–37]. Blastogenic test promotes experimental evidence of the stimulation of cell-mediated immune response components [38], but it does not differentiate among the various biological functions of the lymphocyte subpopulations. Recently, studies have also shown that IFN-ɣ is induced after RB51 vaccination in cattle [39,40], and that immunization with S19 and RB51 stimulate both CD4^+^ and CD8^+^ T-cell responses [41,42]. However, the complete understanding of the immune response triggered by the worldwide used *B*. *abortus* vaccines in cattle is still undefined.

Characterization of protective immunity conferred by *B*. *abortus* vaccines in cattle is critical for the development of new vaccines that are more effective and safer. It may also provide new methods to assess these potential vaccines. Incomplete characterization of *B*. *abortus*-specific T and B lymphocytes subsets preclude a definitive conclusion on the exact role of the immune cell subpopulations in protective response. Furthermore, it is not known whether calves vaccinated with RB51 or S19 have identical profiles and persistence of the immune response. Likewise, there is limited information on the immune response induced by RB51-revaccination. Considering that some countries still use S19 for vaccination of calves, it is important to assess the effects of RB51 revaccination in S19 as well as in RB51 prime-vaccinated animals, since revaccination of adult cattle with RB51 can be used strategically within brucellosis control programs to increase herd immunity, especially in areas of high brucellosis prevalence.

Additionally, as several studies have shown promising results using RB51 and S19 as vaccine vectors for heterologous antigens [21,22,24,25,43–46], the detailed understanding of the immune response generated by these strains could maximize their use as vectors. Therefore, the aims of the present study were to characterize and compare the adaptive immune response of calves vaccinated with *B*. *abortus* S19 or RB51 and revaccinated with RB51.

## Material and Methods

### Locale, animals and experimental design

**Fig 1:**
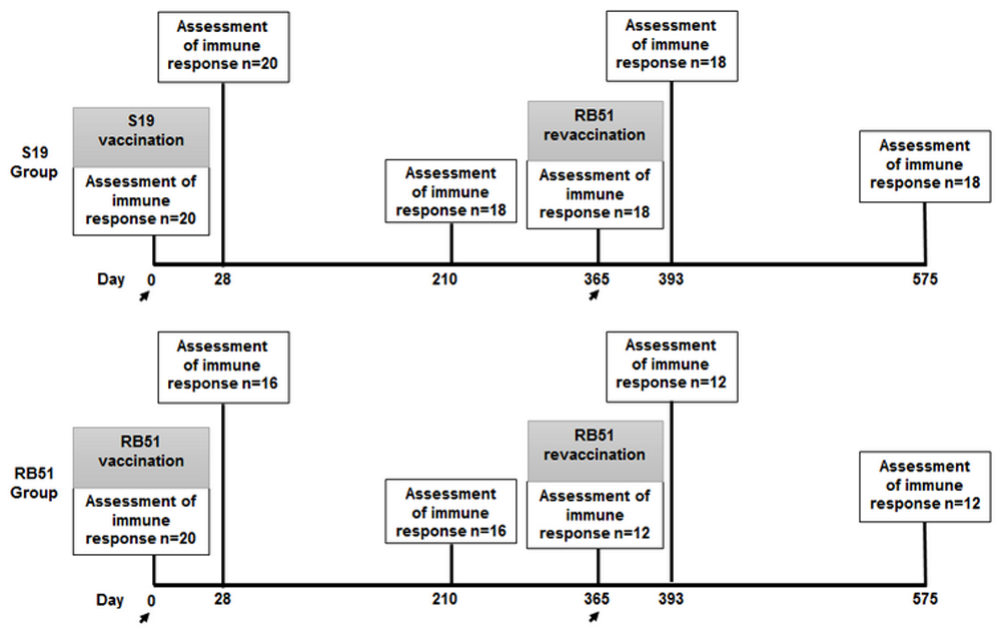
Experimental design. Forty crossbred females calves aged between 4 to 8 months were divided in two experimental groups: group S19—composed of 20 calves vaccinated with S19 vaccine strain (0.6–1.2 x 10^11^ CFU) at day 0 of the experiment; and group RB51—composed of 20 calves vaccinated with RB51 vaccine strain (1.3 x 10^10^ CFU) at day 0 of the experiment. Both groups were revaccinated with RB51 (1.3 x 10^10^ CFU) at day 365 of the experiment. The number of animals tested in each immunological assessment (0,28, 210, 365, 393 and 575) are shown in the rectangles. The days when the vaccinations occurred are highlighted with arrows.

The experiment was conducted in a brucellosis-free dairy herd localized in Baldim, Minas Gerais State, Brazil. Forty crossbred females calves aged between 4 to 8 months were randomly selected and serologically confirmed as brucellosis-negative by rose Bengal agglutination test (RBT), standard tube agglutination test (STAT), and 2-mercaptoethanol test (2ME) [47]. These animals were divided into two experimental groups: group S19—composed of 20 calves vaccinated with S19 vaccine strain at day 0 of the experiment; and group RB51—composed of 20 calves vaccinated with RB51 vaccine strain at day 0 of the experiment (Fig 1). Animals from both groups were revaccinated with RB51 at day 365 of the experiment. The distribution of animals of different ages between groups was random and proportional (mean and median = 5.5 months). All animals were raised semi-intensively and fed a balanced diet of concentrate, mineral salt mixture and pasture.

The experimental design, as well as the number of animals tested at each time point, is shown in Fig 1. For both experimental groups, the evaluation of the immune response was performed at days 0, 28, 210 (7 months), 365 (1 year), 393 (1 year and 28 days) and 575 (1 year and 7 months) after prime vaccination (Fig 1). The characterization of the immune response was performed in cells isolated from peripheral blood, which was collected by venipuncture from all calves at each time point.

### Ethics Statement

Experiments with cattle were carried out in strict accordance Brazilian law on use of animal on research and teaching (Lei n° 11.794/2008) and were approved by the Ethical Committee for the use of Experimental Animals of the Universidade Federal de Minas Gerais, Brazil (CETEA) under protocol 139/2010.

### Vaccines and vaccinations

At day 0 of the experiment, all calves from S19 group were subcutaneously vaccinated with S19 commercial vaccine (0.6–1.2 x 10^11^ CFU) [48]. RB51 group and RB51 revaccinated animals received subcutaneously 1.3 x 10^10^ CFU of viable *B*. *abortus* RB51 [49], at days 0 and 365 of the experiment, respectively. *B*. *abortus* RB51 vaccine strain was provided by Prof. Gehardt. Schurig (Virginia Tech, USA) and the bacterial suspensions for vaccination were prepared according to World Animal Health Organisation (OIE) [2]. Exact doses inoculated were assessed retrospectively [50].

### Monoclonal antibodies (mAbs)

Monoclonal antibodies (mAbs) against cell surface markers, intracellular cytokines, nuclear proteins, immunoglobulins and mAbs that cross-react with bovine cytokines [51] used in the present study are summarized in the Table 1. All mAbs had titration pre-determined before each testing time point.

**Table 1:** Monoclonal antibodies (mAbs) against cell surface markers, intracellular cytokine, nuclear protein or immunoglobulin used in this study.

mAb	Conjugated	Target species	Clone	Host	Isotype	Binding site	Concentration or dilution
Anti-CD4^a^	Alexa Fluor 647 / FITC^g^ / PE^h^	Bovine	CC8	Mouse	IgG2a	Cell surface	0.25 to 0.5 μg/mL
Anti-CD8^a^	Alexa Fluor 647 / FITC / PE	Bovine	CC63	Mouse	IgG2a	Cell surface	0.25 to 0.5 μg/mL
Anti-CD21^a^	FITC / PE	Bovine	CC21	Mouse	IgG1	Cell surface	0.5 to 1.0 μg/mL
Anti-MHC II^a^	FITC	Bovine	IL-A21	Mouse	IgG2a	Cell surface	0.5 to 1.0 μg/mL
Anti-CD25^a^	PE	Bovine	IL-A111	Mouse	IgG1	Cell surface	0.5 to 1.0 μg/mL
Anti-CD45 RO^b^	-	Bovine	GC42A1	Mouse	IgG1	Cell surface	2 to 5 μg/mL
Anti-IgG1^c^	PE-Cy5.5	Mouse		Goat	IgG1	Cell surface	1: 10
Anti-FoxP3^a^	Alexa Fluor 647	Bovine	7627	Human	HuCAL Fab bivalent	Intracellular	1: 25
Anti-IL-4^a^	PE	Bovine	CC303	Mouse	IgG2a	Intracellular	1: 50
Anti-IFN-ɣ^a^	PE	Bovine	CC302	Mouse	IgG1	Intracellular	1: 50
Anti-IL-17A^d^	PE	Human	eBio64DEC17	Mouse	IgG1	Intracellular	0.25 to 0.5 μg/mL
Anti-Granzyme B^e^	PE	Human	351927	Mouse	IgG2a	Intracellular	1 μg/mL
Anti-Perforin^f^	PE	Human	δG9	Mouse	IgG2b	Intracellular	1: 50
Anti-Total IgG^a^	HRP^i^	Bovine	IL-A2	Mouse	IgG1	Ig	1: 5000
Anti-IgG1^a^	HRP	Bovine	IL-A60	Mouse	IgG1	Ig	1: 2500
Anti-IgG2^a^	HRP	Bovine	IL-A73	Mouse	IgG1	Ig	1: 2500
Anti-IL-10	Biotin	Bovine	CC320	Mouse	IgG1	IL-10	3 μg/mL
Anti-IL-10	-	Bovine	CC318	Mouse	IgG2b	IL-10	7 μg/mL

^a^mAb purchased from AbD Serotec (Raleigh, USA).

^b^mAb purchased from VMRD (Pullman, USA).

^c^mAb purchased from Life Technologies (Carlsbad, USA).

^d^mAb purchased from eBioscience (San Diego, USA).

^e^mAb purchased from R&D Systems (Minneapolis, USA).

^f^mAb purchased from BD Pharmingen (San Diego, USA).

^g^fluorescein isothiocyanate (FITC).

^h^phycoerythrin (PE).

^i^horseradish peroxidase.

### Peripheral blood mononuclear cells (PBMC) isolation, culture and immunophenotyping

Peripheral blood mononuclear cells (PBMC) were isolated from heparinized blood samples using Ficoll-Paque density gradient (GE Healthcare, Sweden), as previously described [35]. Cells were cultured in 48-well cell culture plates (1 x 10^6^ cells / well) (Corning, USA) for 6 days at 37°C and 5% CO_2_. Cell viability was monitored by trypan blue staining using light microscopy. Antigen stimulated cultures were incubated with ɣ-irradiated (1.4 x 10^6^ rads) *B*. *abortus* strain 2308 (10^8^ CFU / mL), control cultures with RPMI 1640 (Sigma, USA) and positive control cultures with phytohaemagglutinin-P (PHA-P) (Medicago, Sweden) (5 μg / mL). Brefeldin A (BFA) (Sigma, USA) was added (10 μg / mL) only to wells used for intracellular cytokine immunostaining and these cultures were incubated for an additional period of 4 h in 5% CO_2_ at 37°C. Following incubation, cells were stained with mAbs (Table 1) in four, three or two-color flow cytometric assays according to cell profile investigated (CD4-IFN-ɣ; CD8-IFN-ɣ; CD4-IL-17A; CD8-IL17A; CD4-IL-4; CD8-IL-4; CD8-Perforin; CD8-Granzyme B; CD4-CD45RO; CD8-CD45RO; CD21-CD45RO; CD4-MHCII; CD8-MHCII; CD21-MHCII; CD4-FoxP3-CD25; CD8-FoxP3-CD25). For intracellular cytokine and nuclear protein immunostaining assay the cells were first stained with surface mAbs. Then, PBMC were fixed and permeabilized with permeabilizing buffer (Becton Dickinson, USA), before staining with intracellular mAbs, as previously described [41].

### Cell proliferation assay

PBMC were stained with Carboxyfluorescein Diacetate Succinimidyl Ester (CFSE) (Life Techonologies, USA), according to manufacturer’s instructions. The cells were cultured in 48-well cell culture plates for 6 days (1 × 10^6^ cells / well) at 37°C and 5% CO_2_. Cell viability was monitored by trypan blue staining using light microscopy. Antigen stimulated cultures were incubated with γ-irradiated (1.4 x 10^6^ rads) *B*. *abortus* strain 2308 (10^8^ CFU / mL), control cultures with RPMI 1640 and positive control cultures with PHA-P (5 μg / mL). Following incubation, cells were stained with anti-bovine CD4 and anti-bovine CD8 mAbs conjugated with phycoerythrin (PE) or Alexa-Fluor 647 (Table 1).

### Flow cytometry acquisition and data analysis

**Fig 2:**
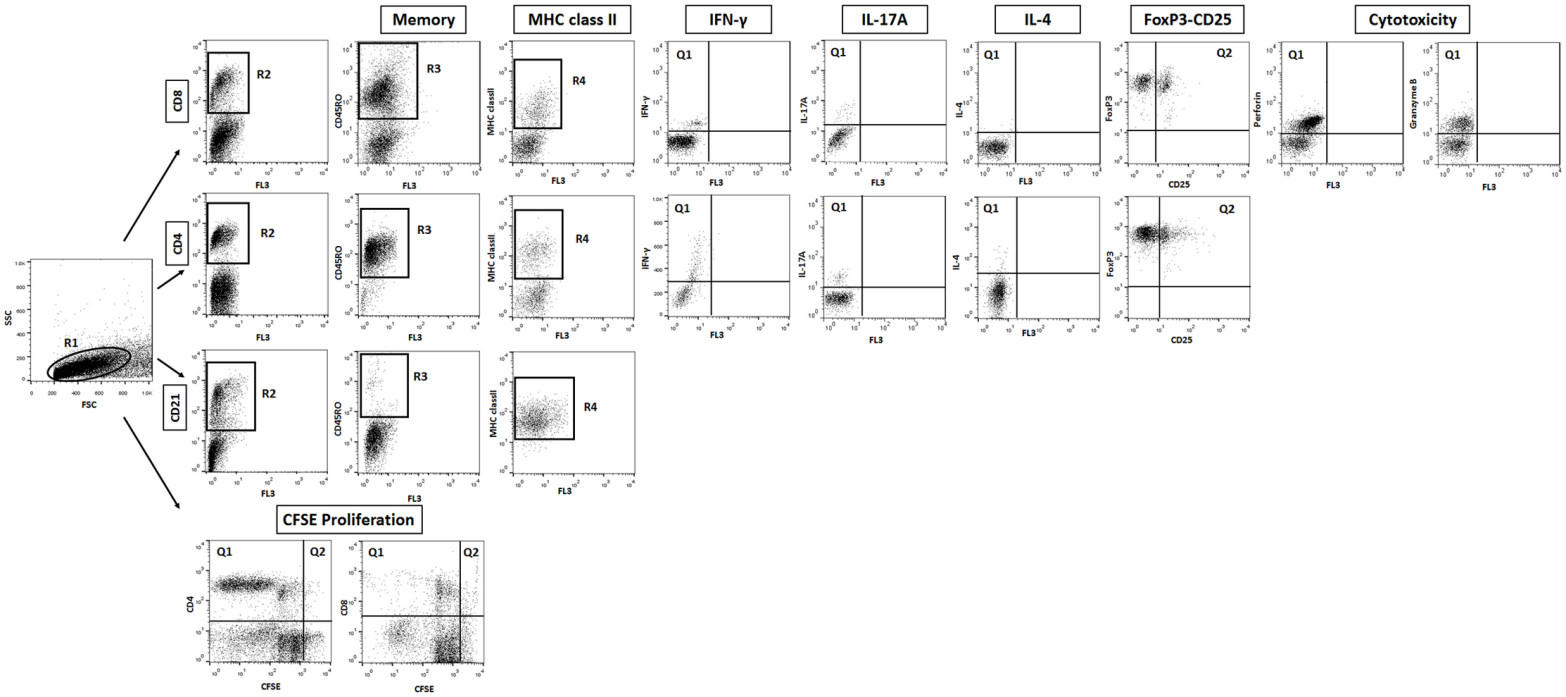
Gating strategies used to select specific leukocytes subpopulations. The lymphocytes were identified as R1 based on their size and granularity flow cytometric features prior to the analysis of CD8^+^, CD4^+^, CD21^+^ lymphocytes subsets identified as R2 and proliferation. Lymphocytes subpopulations expressing the memory marker (CD45RO) were quantified based on R3. The mean of fluorescence intensity of MHC class II on lymphocytes subpopulations were quantified based on R4. Percentage of lymphocytes subsets expressing intracytoplasmic cytokines (IFN-ɣ, IL-17A and IL-4) or cytotoxic markers (perforin and granzyme B) were quantified based on Q1. Percentage of lymphocytes subsets expressing FoxP3-CD25 was determined using quadrant statistics over anti-Foxp3 versus anti-CD25 marker dot plot distribution (Q2). For proliferation assay, the phenotypic analysis was carried to determine the percentage of divided cells using CFSE / anti-bovine surface marker (anti-CD4 or anti-CD8) dot plots.

A minimum of 30,000 cells per sample was analyzed in FACSCalibur (Becton Dickinson, USA) in all assays. The FlowJo 7.6.1 (Tree Star, USA) software was used in all flow cytometry data analysis. Distinct gating strategies were used to analyze the different lymphocyte subpopulations and cytokine-expressing lymphocyte subsets as shown in Fig 2.

Selective analysis of T-cell subsets (CD4^+^ and CD8^+^) and B-cells (CD21^+^) was performed by initially gating the lymphocytes on forward scatter (FSC) versus side scatter (SSC) dot plot distribution (R1), followed by individual or collective analysis on anti-CD4, anti-CD8 and anti-CD21 (R2) (Fig 2). These subpopulation of lymphocytes were then screened for expression of CD45RO (R3), MHC class II (mean of fluorescence intensity) (R4), cytokines (IFN-ɣ, IL-17A and IL-4), FoxP3 and CD25, and cytotoxicity markers (perforin and granzyme B) (Fig 2). For intracellular immunostaining assay following the selection of lymphocytes subset, the frequency of cytokine^+^, FoxP3^+^CD25^+^ or cytotoxicity marker^+^ cells was determined using quadrant statistics over anti-cell surface marker versus anti-cytokine / FoxP3-CD25 / cytotoxicity marker dot plot distribution. The results were expressed as percentages of cytokine^+^, FoxP3^+^CD25^+^ or cytotoxicity marker^+^ cells for different gated leucocytes subpopulations analyzed (CD4^+^ and CD8^+^) (Fig 2).

The level of lymphocyte proliferation was quantified by setting quadrants to segregate the fraction of lymphocytes that have divided and to segregate FL2 / PE or FL4 / Alexa Fluor 647 positive and negative cells based on the negative control immunostaining (Fig 2). Specific lymphocyte proliferation was calculated taking the percentage of lymphocytes that express CD4 or CD8 that proliferated divided by the percentage of the surface marker of interest expressing-lymphocytes [(Q1 / Q1 + Q2) × 100].

### IL-4, IL-6, IL-10 and IFN-ɣ detection by ELISA

Supernatants of 6-day-old cultures were tested for the presence of IL-4, IL-6, IL-10 and IFN-ɣ by antigen-capture enzyme-linked immunosorbent assays (ELISA). The assays were performed according to manufacturer's recommendations (Thermo Fisher Scientific, USA) for IL-4, IL-6 and IFN-ɣ. The ELISA for detection of IL-10 was performed according Kwong et al. [52], using anti-bovine IL-10 (clone CC318) (AbD Serotec, USA) as capture antibody, anti-bovine IL-10-biotin (clone CC320) (AbD Serotec, USA) as detection antibody (Table 1) and recombinant bovine IL-10 (Kingfisher, USA) for developing a standard curve.

### Serologic assays

Sera collected at each time point (0, 28, 210, 365, 393 and 575) were centrifuged, separated in aliquots and stored at -20°C. To detect anti-S19 and anti-RB51 antibodies two kinds of antigens, whole-cell [53] and lysed heat-killed [54] antigens, produced using *B*. *abortus* S19 and *B*. *abortus* RB51 strains were used in an indirect ELISA (I-ELISA). *B*. *abortus* S19 strain was obtained in lyophilized form from USDA National Veterinary Services Laboratory, Ames, Iowa, United States of America (USA). *B*. *abortus* S19 antigens were used to test serum samples from S19 group. *B*. *abortus* RB51 antigens were used to test serum samples from RB51 group. Serum samples from S19 group collected at days 365, 393 and 575 were also tested using the two kinds of antigens (whole-cell and lysed heat-killed) produced from *B*. *abortus* RB51. All I-ELISA assays were performed similarly. Briefly, the antigens were adsorbed onto polystyrene plates (Nunc Maxisorp, Thermo Fisher, USA) at a concentration of 1.0 μg / well in bicarbonate buffer (0.06 M, pH 9.6, Sigma Aldrich, USA) at 4–8°C overnight. Plates were blocked with phosphate buffered saline (0.01 M, pH 7.4, all from Merck, Germany) with 5% of non-fat dry milk at 37°C for 1 h. Serum samples at 1: 100 (S19 group) and 1: 50 (RB51 group) dilution were added to the wells in duplicate and incubated at 37°C for 1 h. The best dilution of sera for each group was previously determined in order to obtain the greater difference from sera of non-vaccinated calves. Isotype-specific mouse anti-bovine horseradish peroxidase conjugates (Total IgG, IgG1 and IgG2) (Table 1) were added and the plates were incubated at 37°C for 1 h. The substrate solution, 3.3’, 5.5’-tetrametilbenzidina-peroxidase (TMB) (Sigma, USA) was added and the reaction stopped using equal volumes of 0.6 N sulfuric acid (Merck, Germany). The absorbance of the developed color was measured at 450 nm. Besides I-ELISA, sera from both groups in all time points were also tested by Bengal agglutination test (RBT), standard tube agglutination test (STAT), and 2-mercaptoethanol test (2ME) [47].

### Quantitative real time reverse transcriptase-polymerase chain reaction (qRT-PCR) for IL-10 and TGF-β detection

After six days of culture, total RNA extraction from PBMC samples was carried out with Trizol Reagent (Life Technologies, Carlsbad, USA) following the manufacturer’s instructions. cDNA strands were synthesized from 1.5 μg of total RNA using the TaqMan Reverse Transcription kit (Applied Biosystems, Foster City, SA) with oligodT primers according to the manufacturer’s instructions.

Primers used to amplify glyceraldehyde 3-phosphate dehydrogenase (GAPDH) (F- 5’ ATGGTGAAGGTCGGAGTGAACG 3’ and R- 5’ TGTAGTGAAGGTCAATGAAGGGGTC 3’), IL-10 (F- 5’ TGCTGGATGACTTTAAGGG 3’ and R- 5’ AGGGCAGAAAGCGATGACA 3’) and TGF-β (F- 5’ GCCATCCGCGGCCAGATTTTGT 3’ and R- 5’ AGGCTCCGTTTCGGCACTT 3’) were designed from sequences deposited in GenBank, with the help of Primer Express 3.0 Software (Applied Biosystems, Foster City, USA). GAPDH gene was chosen as a housekeeping / control gene. Quantitative RT-PCR was performed using SYBR Green PCR Master Mix (Applied Biosystems 7500 Real Time PCR System, Foster City, USA). qPCR was carried out in a final volume of 25 μL containing 1 μM of forward and reverse primers, SYBR Green PCR Master Mix, and cDNA diluted at 1: 3. The efficiency of each pair of primers was evaluated by serial dilution of cDNA according to the protocol developed by Applied Biosystems. Melting point analysis was done after the last cycle to verify the amplification specificity. In order to evaluate gene expression, two replicate analyses were performed and the amount of target RNA was normalized with respect to the control (housekeeping) gene GAPDH and expressed according to relative curve quantitation of gene expression method [55]. The results are expressed as fold-difference of expression levels (fold-change).

### Statistical analysis

The normalization of the data from subtraction of values of Ag-stimulated culture by values of control cultures was adopted to keep the homogeneity of the variance (homoscedasticity), since this was a long-time experiment. This procedure was performed for all flow cytometry and ELISA for cytokines detection data.

Data were first tested for normality and variance of data sets using Epicalc package [56] of R software version 3.0.1 [57]. Considering the nonparametric nature of data from flow cytometry and ELISA for cytokines detection, analyses among days within the same vaccination regimen were performed by Skillings-Mack test followed by Wilcoxon signed rank test [58], using Skillings.Mack [59] and Stats packages of R software [57], respectively. Analyses between vaccination regimens within the same day were performed by Mann-Whitney test, also using the Stats package of R software [57]. For I-ELISA data, analyses among days within the same vaccination regimen were performed by one-way ANOVA followed by paired t-test (Graphpad PRISM 5.0, GraphPad Software, USA), considering its parametric nature. Significance was defined in all cases at P < 0.05 [60].

## Results

The main focus of results was comparisons between: pre-vaccinated and vaccinated animals (day 0 vs. 28); peak and mid-term vaccination immune responses (day 28 vs. 210 and day 28 vs. 365); mid-term vaccination immune response and revaccination (day 365 vs. 393); and peak and mid-term revaccination immune response (day 393 vs. 575).

### Immune response induced following S19 or RB51 vaccination

#### S19 and RB51 vaccination significantly increase the proliferation of antigen-specific CD4^+^ and CD8^+^ T-cells

**Fig 3:**
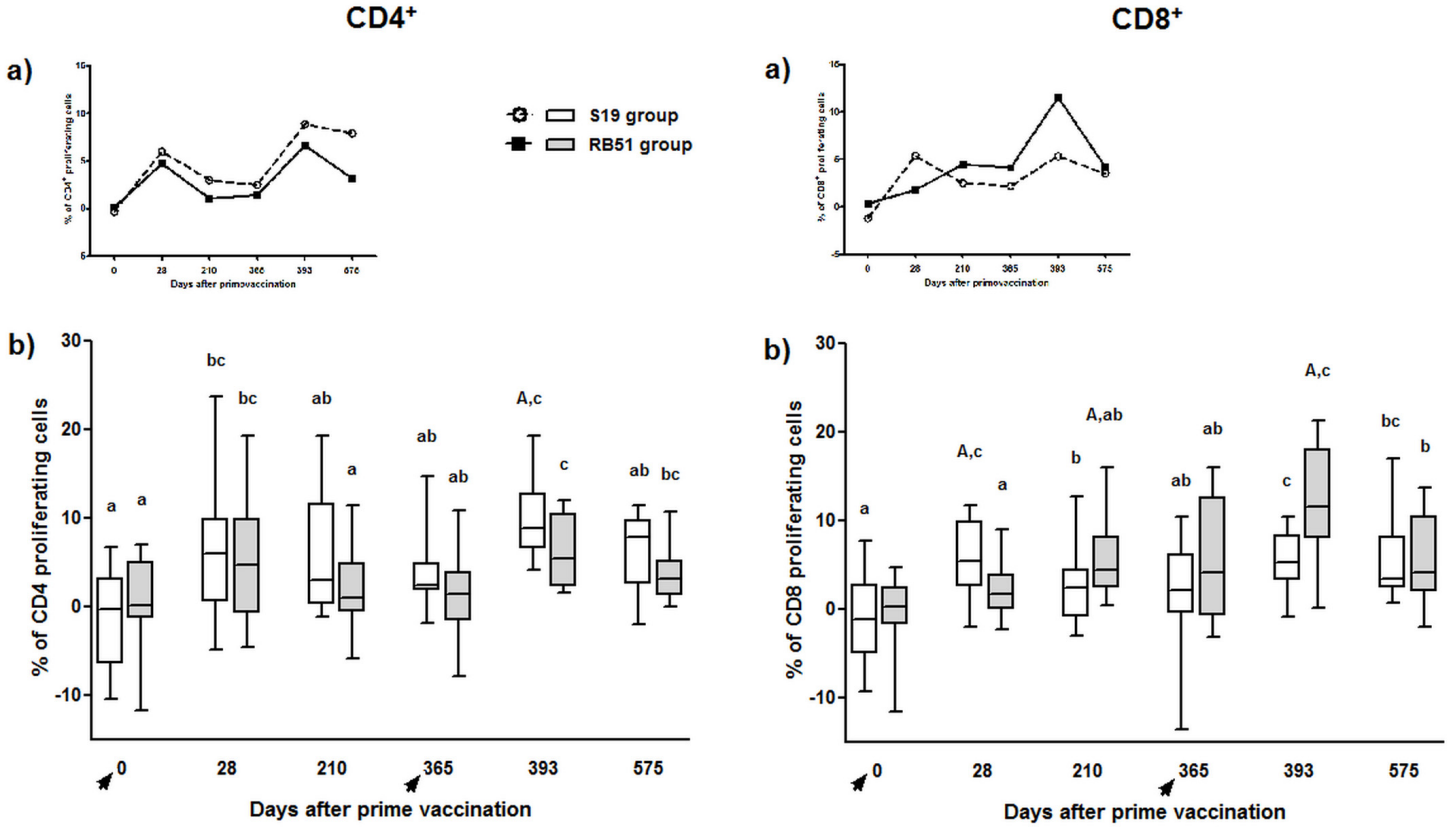
CFSE proliferation analysis of CD4^+^ and CD8^+^ T-cells subsets in peripheral blood mononuclear cells of S19 and RB51 prime vaccinated, and RB51 revaccinated cattle upon *in vitro* stimulation with ɣ-irradiated *B*. *abortus* 2308. Tendency (median) (a) and box plot (median, first and third quartiles) (b) charts of the results. Whiskers show the lower and upper 1.5 interquartile range. Vaccinations were indicated by arrows. Significant differences (P < 0.05) between vaccination regimens (on same day) are indicated by uppercase letters (Mann-Whitney-test), and lowercase letters indicate statistical differences between days in same group (Skillings Mack test followed by Wilcoxon signed rank test).

Comparison between pre-vaccinated animals (day 0) and calves at 28 days post-vaccination showed a significant increase in proliferation of antigen-specific CD4^+^ and CD8^+^ T-cells in both, S19 and RB51-vaccinated calves (Fig 3). However, on days 210 and 365 following S19 prime vaccination, a decrease in CD8^+^ T-cell proliferation was observed compared to day 28, in which S19 induced a superior CD8^+^ T-cell proliferation than RB51. Likewise, on day 210 there was a significant decline in CD4^+^ T-cell proliferation compared to day 28 in RB51 group.

#### S19 vaccination significantly increased CD8^+^Granzyme B^+^ T-cells, whereas RB51 vaccination significantly increased both CD8^+^Granzyme B^+^ and CD8^+^Perforin^+^ T-cells

**Fig 4:**
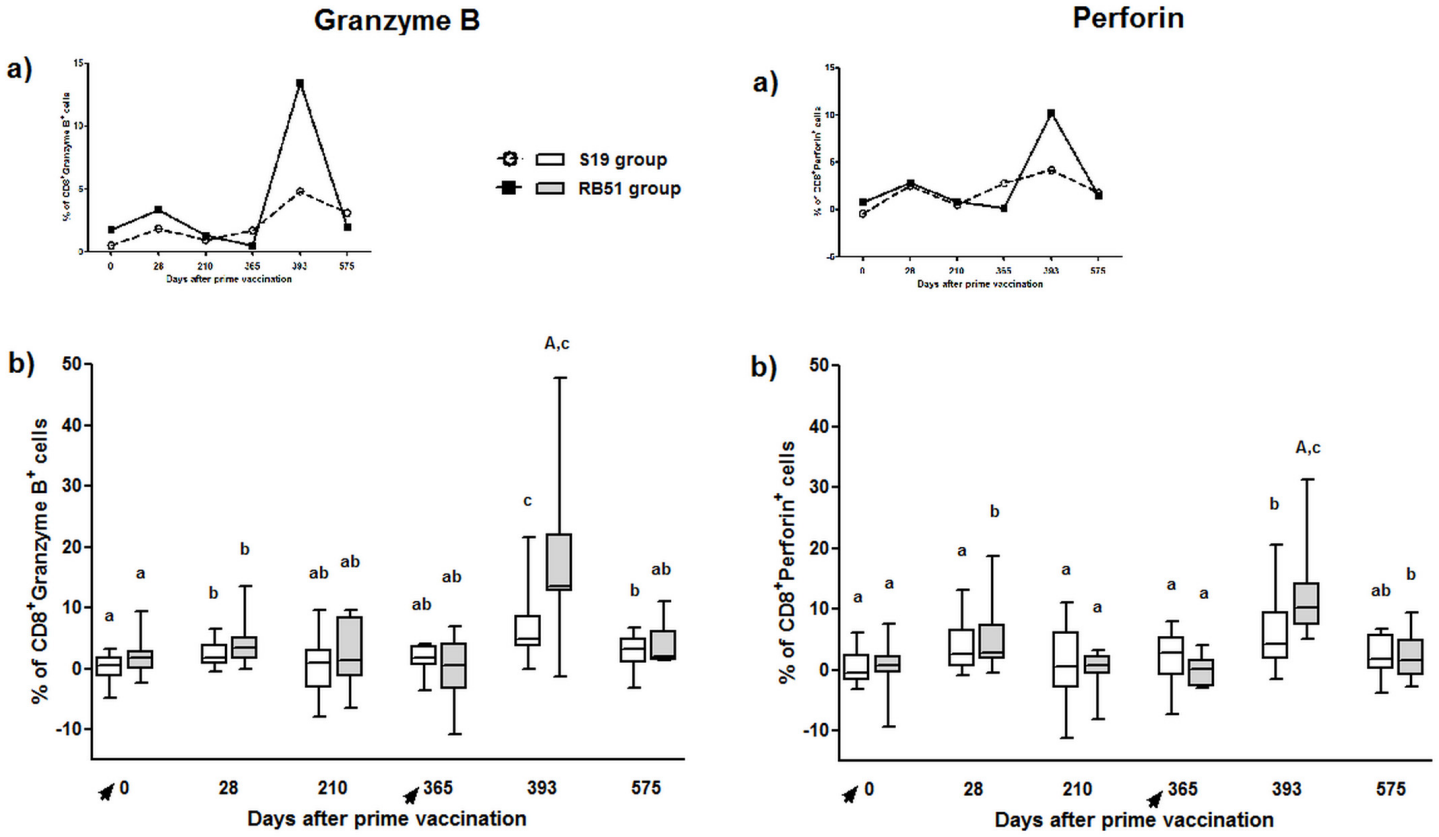
Granzyme B and perforin-expressing CD8^+^ T-cells in peripheral blood mononuclear cells of S19 and RB51 prime vaccinated, and RB51 revaccinated cattle upon *in vitro* stimulation with ɣ-irradiated *B*. *abortus* 2308. Tendency (median) (a) and box plot (median, first and third quartiles) (b) charts of the results. Whiskers show the lower and upper 1.5 interquartile range. Vaccinations were indicated by arrows. Significant differences (P < 0.05) between vaccination regimens (on same day) are indicated by uppercase letters (Mann-Whitney-test), and lowercase letters indicate statistical difference between days in same group (Skillings-Mack test followed by Wilcoxon signed rank test).

Comparison between pre-vaccinated calves (day 0) and animals 28 days after vaccination showed that S19 induced CD8^+^Granzyme B^+^ T-cells and RB51 induced CD8^+^Granzyme B^+^ and CD8^+^Perforin^+^ T-cells (Fig 4). However, for RB51 vaccinated animals the levels of CD8^+^Perforin^+^ T-cells significantly decreased on days 210 and 365 in comparison to day 28.

#### CD4^+^ T-cells are the main source of IFN-ɣ following S19 or RB51 vaccination

**Fig 5:**
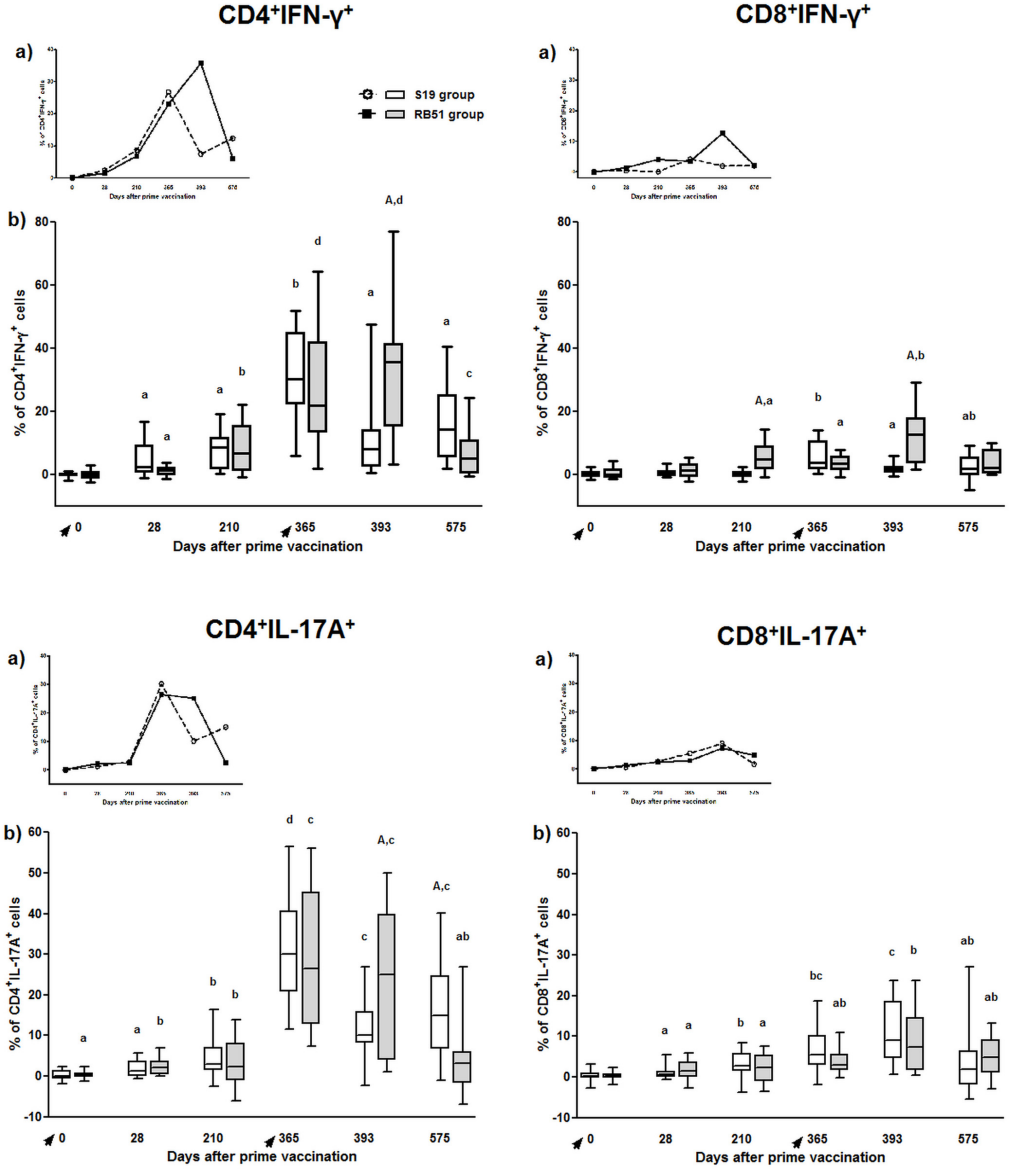
IFN-ɣ and IL-17A production by CD4^+^ and CD8^+^ T-cell subsets in peripheral blood mononuclear cells of S19 and RB51 prime vaccinated, and RB51 revaccinated cattle upon *in vitro* stimulation with ɣ-irradiated *B*. *abortus* 2308. Tendency (median) (a) and box plot (median, first and third quartiles) (b) charts of the results. Whiskers show the lower and upper 1.5 interquartile range. Vaccinations were indicated by arrows. Significant differences (P < 0.05) between vaccination regimens (on same day) are indicated by uppercase letters (Mann-Whitney-test), and lowercase letters indicate statistical difference between days in same group (Skillings Mack test followed by Wilcoxon signed rank test).

S19 and RB51 vaccination induced the production of significant levels of IFN-ɣ, whose main source was CD4^+^ T-cells (Fig 5). Comparison between pre-vaccinated calves (day 0) and the same group 28 days after vaccination showed a significant increase in CD4^+^IFN-ɣ^+^ for both vaccination regimens. In comparison to day 28, CD4^+^IFN-ɣ^+^ T-cells also showed a significant increase on day 365 and on days 210 and 365 for S19 group and RB51 group, respectively. Significant levels of CD8^+^IFN-ɣ^+^ T-cells were induced later after vaccination, on day 365 and 210, for S19 and RB51, respectively.

#### S19 and RB51vaccination induced significant levels of CD4^+^IL-17A^+^ and CD8^+^IL-17A^+^, being CD4^+^ T-cells the main source of IL-17A

S19 and RB51 vaccination induced the production of significant levels of IL-17A, whose main source was CD4^+^ T-cells (Fig 5). Comparison between calves at day 0 and the same group 28 days after vaccination showed a significant increase in CD4^+^ and CD8^+^ T-cells producing-IL-17A^+^ for both vaccination regimens. However, production of IL-17A increased significantly after S19 and RB51 vaccination peaking one year after vaccination (day 365) (Fig 5) only for CD4^+^ T-cells.

#### S19 and RB51 vaccination induced IFN-ɣ responses

**Fig 6:**
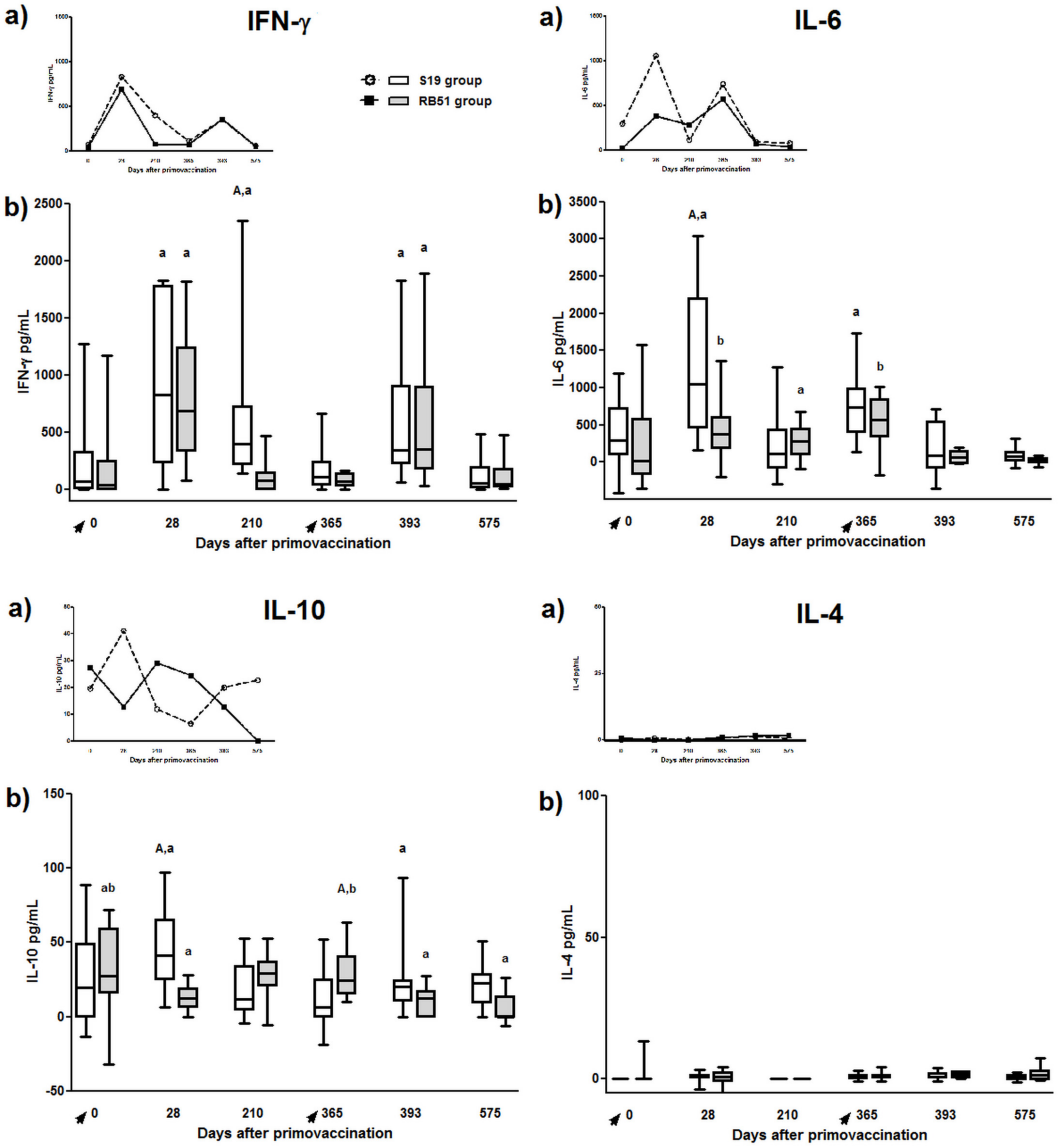
IFN-ɣ, IL-6, IL-4 and IL-10 accumulated in cell culture supernatant of peripheral blood mononuclear cells of S19 and RB51 prime vaccinated, and RB51 revaccinated cattle upon *in vitro* stimulation with ɣ-irradiated *B*. *abortus* 2308. Tendency (median) (a) and box plot (median, first and third quartiles) (b) charts of the results. Whiskers show the lower and upper 1.5 interquartile range. Vaccinations were indicated by arrows. Significant differences (P < 0.05) between vaccination regimens (on same day) are indicated by uppercase letters (Mann-Whitney-test), and lowercase letters indicate statistical difference between days in same group (Skillings Mack test followed by Wilcoxon signed rank test).

Significant antigen-specific IFN-ɣ responses were observed in calves vaccinated with S19 or RB51 on 28 day after vaccination compared to pre-vaccinated animals (day 0) (Fig 6). However, only S19 vaccinated animals presented significant IFN-ɣ accumulation in culture supernatant seven months (day 210) after immunization compared to pre-vaccinated animals (day 0). In addition, the antigen-specific IFN-ɣ responses of the S19 and RB51 vaccinated cattle decreased one year (day 365) post-vaccination compared to animals on day 28.

#### S19 or RB51 vaccination did not induce significant levels of IL-4 nor CD4^+^IL-4^+^ or CD8^+^IL-4^+^ cell response

No significant levels of IL-4 were observed in cell culture supernatant on any time for both vaccination regimens or between the vaccination regimens at the same time point (Fig 6). Likewise, there was no significant difference in the intracellular expression of IL-4 by CD4^+^ or CD8^+^ T-cells among any time point for both vaccination regimens or between the vaccination regimens at the same time point (data not shown).

#### S19 induced higher IL-6 secretion than RB51 following vaccination

Following vaccination with S19 or RB51 there was a significant increase in IL-6 production (day 0 vs. 28), which was higher in S19-vaccinated calves than RB51-vaccinated animals (Fig 6). For RB51 prime-vaccinated group, the levels of IL-6 decreased significantly on day 210 but remained high on day 365, compared to day 28. Similarly, for S19 group production of IL-6 was still high on day 365, compared to day 28.

#### Only cells from calves vaccinated with S19 produced significant levels of IL-10 following vaccination

Comparison between days 0 and 28 revealed that cells from calves vaccinated with S19, but not vaccinated with RB51, produced significant levels of IL-10 (Fig 6). This IL-10 secretion for S19 group significantly decreased on days 210 and 365 compared to day 28. RB51-prime vaccinated animals exhibited an increase in IL-10 production only on day 365 compared to day 28.

#### S19 and RB51 vaccination induced CD4^+^CD45RO^+^ and CD8^+^CD45RO^+^ cells, but only S19 stimulated the development of CD21^+^CD45RO^+^ cells

**Fig 7:**
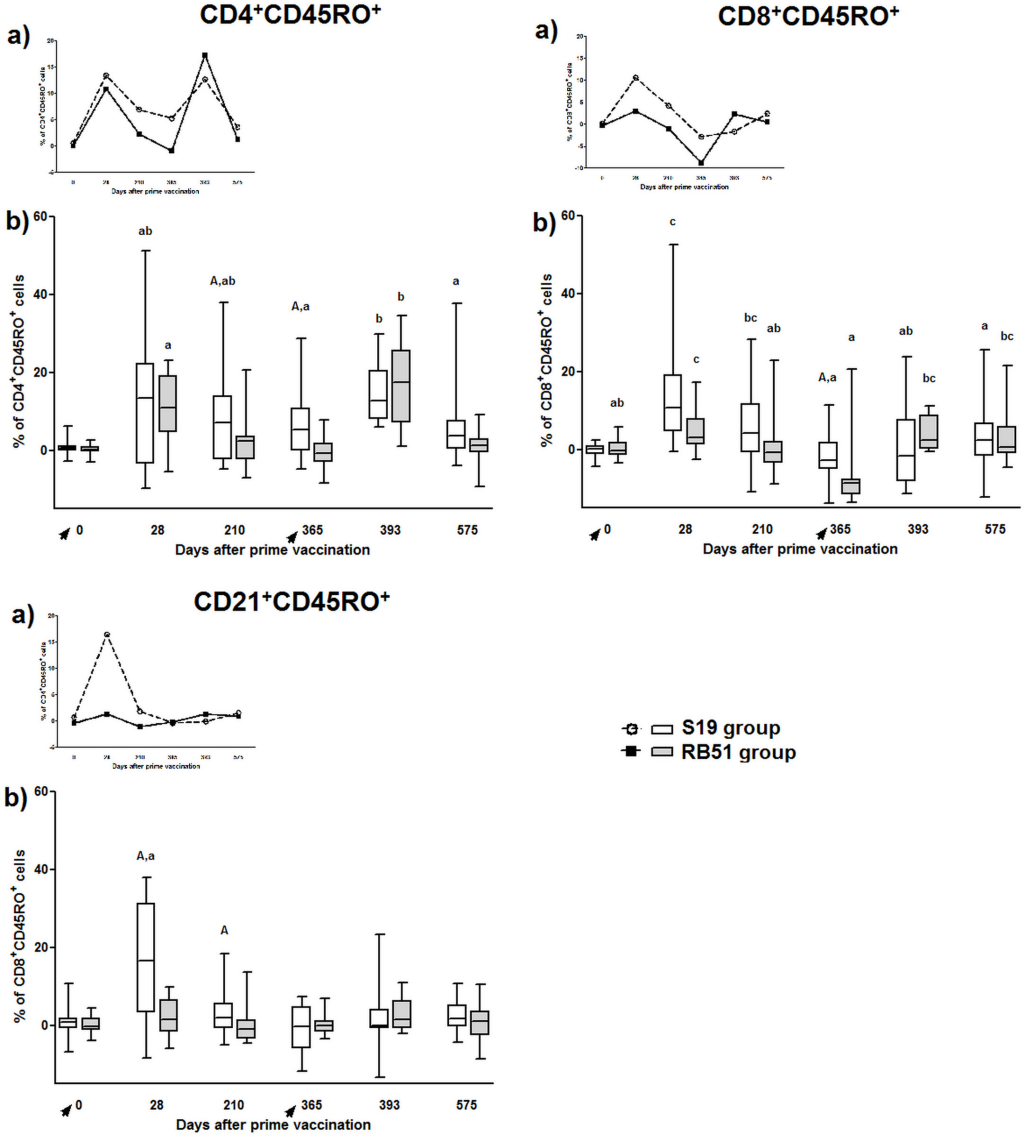
Subsets of memory (CD45RO^+^) lymphocytes in peripheral blood mononuclear cells of S19 and RB51 prime vaccinated, and RB51 revaccinated cattle upon *in vitro* stimulation with ɣ-irradiated *B*. *abortus* 2308. Tendency (median) (a) and box plot (median, first and third quartiles) (b) charts of the results. Whiskers show the lower and upper 1.5 interquartile range. Vaccinations were indicated by arrows. Data of CD45RO^+^ lymphocytes are shown for CD4^+^, CD8^+^ and CD21^+^ cells. Significant differences (P < 0.05) between vaccination regimens (in same day) are indicated by uppercase letters (Mann-Whitney-test), and lowercase letters indicate statistical difference between days in same group (Skillings Mack test followed by Wilcoxon signed rank test).

Assessment of immune response 28 days after S19 and RB51 vaccination showed a substantial increase in CD4^+^CD45RO^+^ and CD8^+^CD45RO^+^ T-cells compared to pre-vaccinated animals (day 0) (Fig 7). On day 210 post-vaccination, only S19 group still exhibited high levels of CD8^+^CD45RO^+^ T-cells. However on day 365 after vaccination both groups showed a significantly reduction in CD8^+^CD45RO^+^ T-cells compared with day 28, being this reduction higher in RB51 prime-vaccinated animals.

After S19 prime-vaccination, the level of CD4^+^CD45RO^+^ T-cells significantly increased on day 28 and was kept high until one year post-vaccination. RB51 vaccinated calves, although having a significant increase of CD4^+^CD45RO^+^ T-cells on day 28 showed a significant decrease of these cells on days 210 and 365 (Fig 7). Compared with RB51, S19 group showed significant higher levels of CD4^+^CD45RO^+^ T-cells on days 210 and 365 post-vaccination.

Vaccination with S19, but not RB51, induced significant levels of CD21^+^CD45RO^+^ B-cells 28 days after immunization. CD21^+^CD45RO^+^ B-cells were significantly higher in S19 prime-vaccinated animals on days 28 and 210 post-vaccination comparing to RB51 prime-vaccinated animals at the same days.

#### IgG1 was the main antibody class produced following S19 and RB51 vaccination

**Fig 8:**
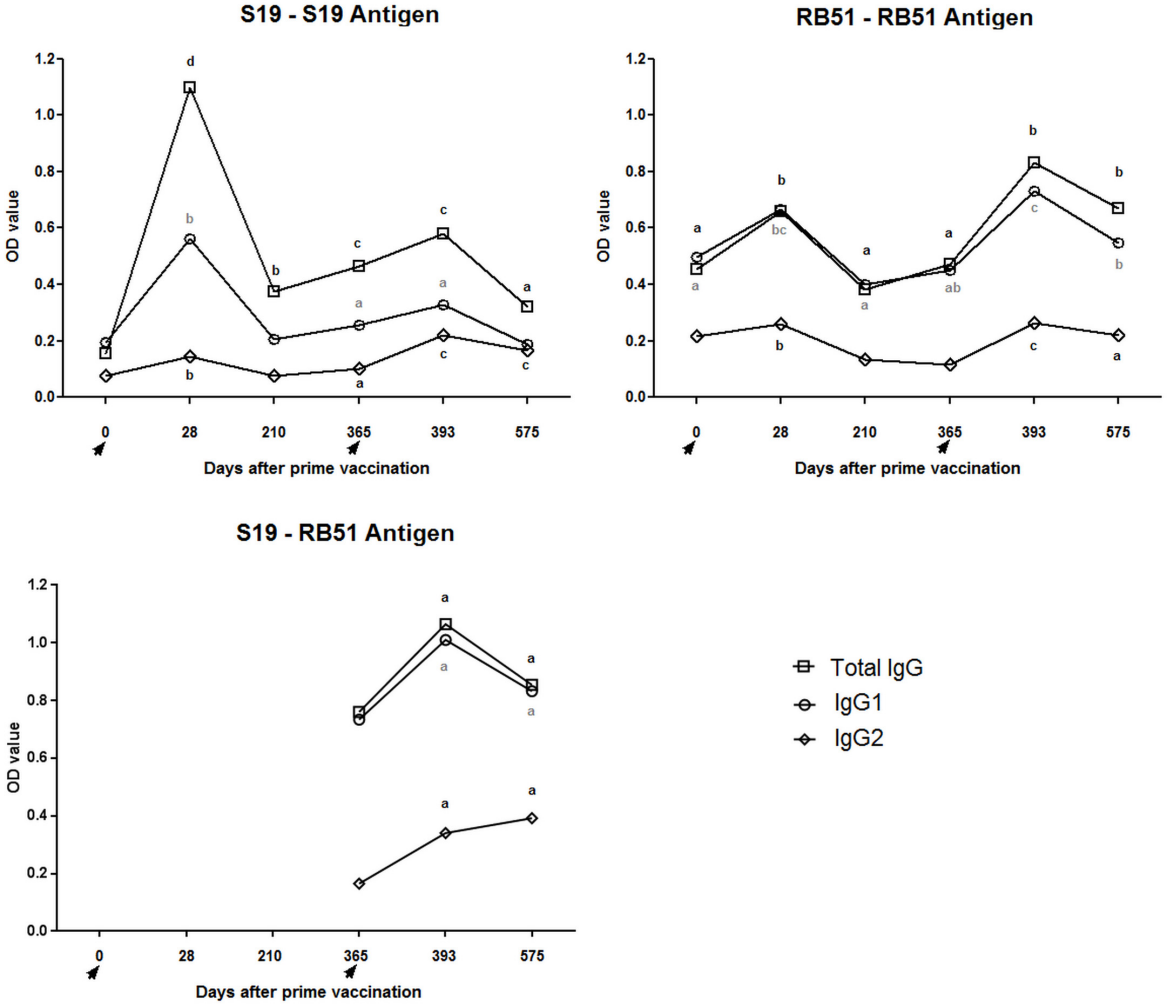
Antibody profile of S19 and RB51 prime vaccinated, and RB51 revaccinated cattle measured by I-ELISA using S19 and RB51 whole-cell antigens. The results are expressed as mean. Data for total IgG, IgG1 and IgG2 are shown. Vaccinations were indicated by arrows. Lowercase letters indicate statistical difference between days on same group (one-way ANOVA followed by paired t-test).

In the two vaccination regimens and throughout all time points assessed there was a predominance of the IgG1 isotype over IgG2 (Fig 8). S19 as well as RB51 prime-vaccination induced significant levels of total IgG, IgG1 and IgG2 in cattle (day 0 vs. 28). Comparisons between day 28 and days 210 and 365 showed a significant decrease in all antibody isotypes tested for both, S19 and RB51 groups.

### Immune response induced following RB51 revaccination

#### RB51 revaccination significantly increased the proliferation of antigen-specific CD4^+^ and CD8^+^ T-cells

After RB51 revaccination (day 393), for both groups (S19 and RB51), a significant increase in CD4^+^ and CD8^+^ T-cell proliferation was observed in comparison to days 0 and 365 (Fig 3). Nevertheless, following RB51 revaccination (day 393), S19 prime-vaccinated animals exhibited higher CD4^+^ T-cell proliferation compared to RB51 prime-vaccinated animals, whereas RB51 prime-vaccinated group showed significant higher CD8^+^ T-cell proliferation compared to S19 group. Comparison between day 393 (peak of immune response after revaccination) and day 575 showed a decrease in CD4^+^ T-cell proliferation for S19 group and a decrease in CD8^+^ T-cell proliferation for RB51 group. Comparison between day 393 and day 575 also showed a non-significant decrease in CD4^+^ T-cell and CD8^+^ T-cell proliferation in groups S19 and RB51, respectively.

RB51 revaccination significantly increased CD8^+^Granzyme B^+^ and CD8^+^Perforin^+^ T-cells. Following RB51 revaccination, on day 393, both vaccination regimens exhibited significant increase in CD8^+^Granzyme B^+^ and CD8^+^Perforin^+^ T-cells in comparison to day 365, even though RB51 prime-vaccinated animals had shown a higher level of CD8^+^Granzyme B^+^ and CD8^+^Perforin^+^ T-cells than S19 group on day 393 (Fig 4). Compared to day 393, RB51 group exhibited lower levels of CD8^+^Granzyme B^+^ and CD8^+^Perforin^+^ T-cells on day 575, whereas S19 group showed significant lower levels only of CD8^+^Granzyme B^+^ on day 575.

#### RB51 revaccination induced a strong IFN-ɣ responses

After RB51 revaccination (day 393), both vaccination regimens exhibited a significant increase in IFN-ɣ responses compared to day 365. On day 575, a decrease in IFN-ɣ responses was observed in both groups compared to the response on day 393 (Fig 6).

#### CD4^+^IFN-ɣ^+^ and CD8^+^IFN-ɣ^+^ T-cell responses were significantly higher in RB51-prime-vaccinated animals after RB51 revaccination, compared to S19-prime vaccinated cattle

After RB51 revaccination (day 365 vs 393), CD4^+^IFN-ɣ^+^ T-cells decreased only in S19 group. Moreover, comparison between the two vaccination regimens on day 393 showed higher levels of CD4^+^IFN-ɣ^+^ T-cells and CD8^+^IFN-ɣ^+^ T-cells in RB51 prime-vaccinated group (Fig 5). Seven months after RB51 revaccination (day 575), only RB51 group exhibited decrease in CD4^+^IFN-ɣ^+^ T-cells compared to day 393. Furthermore, after RB51 revaccination (day 393), only RB51 prime-vaccinated animals increased IFN-ɣ production by CD8^+^ T-cells, in comparison to animals before revaccination (day 365). In contrast, comparison between days 365 and 393 for S19 group showed a decrease in CD8^+^IFN-ɣ^+^ T-cells. The peak of CD8^+^IFN-ɣ^+^ T-cells was observed on day 365 and on day 393 for S19 and RB51 group, respectively. In addition, RB51 group exhibited higher levels of CD8^+^IFN-ɣ^+^ T-cells than S19 group on days 210 and 393. Seven months after RB51 revaccination (day 575), only RB51 group exhibited decrease in CD8^+^IFN-ɣ^+^ T-cells compared to day 393.

#### RB51-prime-vaccinated animals induced significantly higher levels of CD4^+^IL-17A^+^ than S19-prime vaccinated cattle after RB51 revaccination

Only for S19 group, on day 393, CD4^+^IL-17A^+^ T-cells showed lower levels than on day 365 (Fig 5). At the last immune assessment (day 575), S19 group showed higher levels of CD4^+^IL-17A^+^ T-cells compared to RB51 group at the same day. Although presenting lower levels than CD4^+^ T-cells, CD8^+^ T-cells also showed significant increase in IL-17A production after S19 and RB51 vaccination. For CD4^+^ IL-17A^+^T-cells, there was a significant increase on day 393 for both groups compared to day 28.

#### RB51 revaccination did not induce significant levels of IL-4 nor CD4^+^IL-4^+^ or CD8^+^IL-4^+^ cell response

No significant levels of IL-4 were observed in cell culture supernatant on any time after RB51 revaccination for both vaccination regimens or between the vaccination regimens at the same time point (Fig 6). Likewise, there was no significant difference in the intracellular expression of IL-4 by CD4^+^ or CD8^+^ T-cells among any time point for both vaccination regimens or between the vaccination regimens at the same time point after revaccination (data not shown).

#### After RB51 revaccination, IL-6 levels decreased in both vaccination regimens, whereas secretion of IL-10 increase only in S19 prime vaccinated cattle

IL-6 response significantly decreased after revaccination with RB51 for both S19 and RB51-prime-vaccinated cattle (day 365 vs. 393). This reduction remained, for both vaccination regimens, when day 393 and 575 were compared (Fig 6).

As well as observed following prime vaccination, only S19 group exhibited a significant increase in IL-10 secretion after RB51 revaccination (day 365 vs. 393), which decreased seven months after revaccination (day 575). On the other hand, RB51 group showed a significant decrease in IL-10 levels following the revaccination (day 365 vs. 393). On day 575, IL-10 production was not significantly different from day 393, for RB51 group.

#### Following RB51 revaccination, S19 and RB51 prime vaccinated animals showed significant induction in CD4^+^CD45RO^+^ T-cells, but only RB51 group exhibited increase in CD8^+^CD45RO^+^ T-cells

After RB51 revaccination (day 393), the level of CD4^+^CD45RO^+^ T-cells in RB51 group significantly increased compared to day 365, but it decreased again between day 393 and 575 (Fig 7). For S19 group the level of CD4^+^CD45RO^+^ T-cells increased by RB51 revaccination (365 vs 393) and decreased between day 393 and 575 (Fig 7). Following RB51 revaccination (day 393), only RB51 prime-vaccinated group had a significant increase in CD8^+^CD45RO^+^ T-cells compared to animals before revaccination (day 365). The induction of CD8^+^CD45RO^+^ T-cells in RB51 group was still higher on day 575, compared to day 393. After RB51 revaccination, there was no induction of CD21^+^CD45RO^+^ B-cells in both groups.

#### IgG1 was the main antibody class produced also after RB51 revaccination

After RB51 revaccination (day 393), as well as observed following prime vaccination, there was a predominance of the IgG1 isotype over IgG2 (Fig 8). However, after revaccination, RB51 and S19 group, tested with RB51 antigen, exhibited a significant increase in all IgG isotypes tested compared to day 365. S19 group tested with S19 antigen only showed an increase of IgG2 after RB51 revaccination (day 365 vs. 393). Comparison between days 393 and 575 showed a decrease in IgG1 and IgG2 for RB51 group. Likewise, S19 group tested with S19 antigen exhibited a decrease of total IgG and IgG1 between days 393 and 575. However, the levels of all IgG isotypes tested were maintained in animals from S19 group tested with RB51 antigen between days 393 and 575.

#### Immune response following S19 or RB51 vaccination, as well as after RB51 revaccination was predominantly Th1

**Fig 9:**
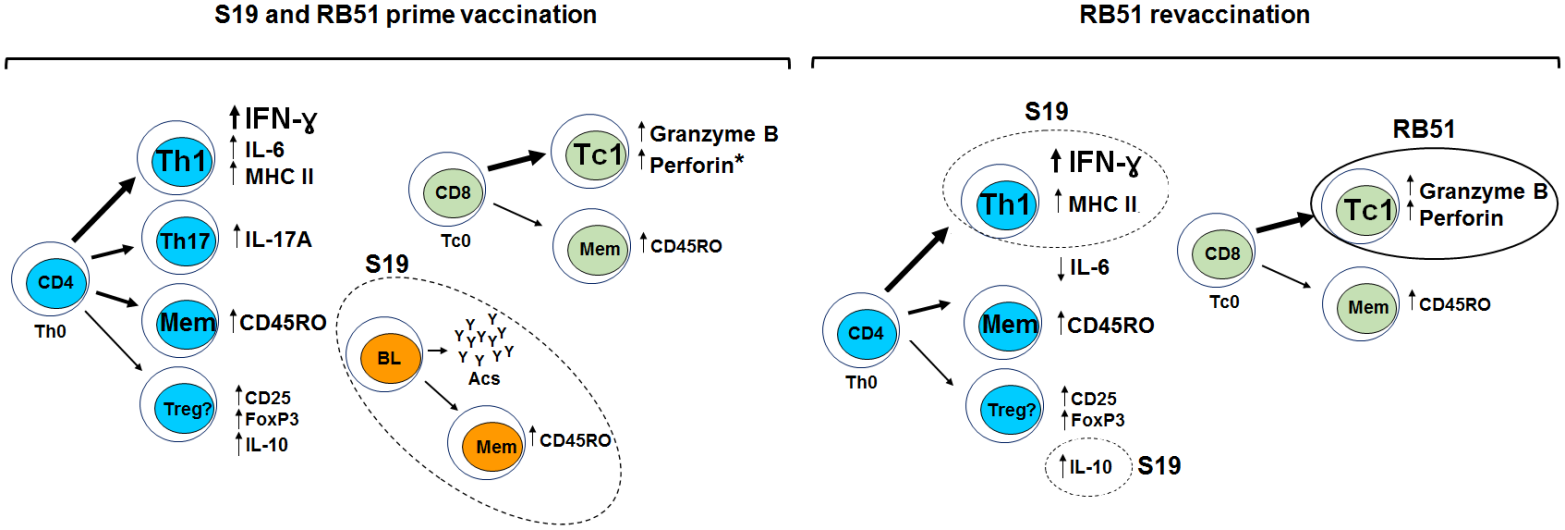
The key mechanisms of adaptive immune system induced after S19 or RB51 prime vaccination and following RB51 revaccination in cattle. Font and arrow sizes indicate the intensity of induction of the mechanism after vaccination or revaccination. Dash lines highlight are the mechanisms dominants in S19 group, whereas solid lines highlight the mechanisms dominants in RB51 group. Asterisk indicate the immunological parameter significantly induced only following RB51 vaccination.

The key mechanisms of adaptive immune system induced after S19 or RB51 prime vaccination and following RB51 revaccination in cattle are summarized in the Fig 9. Immune response after S19 or RB51 vaccination, as well as after RB51 revaccination is chiefly Th1, with great participation of IFN-ɣ, IL-6, CD4^+^IFN-ɣ^+^ T-cells, cytotoxic CD8^+^ T-cells, CD4^+^ and CD8^+^ memory cells (Figs 4, 5, 6, 7 and 9).

## Discussion

So far, it is not established whether calfhood vaccination with S19 or RB51 induces equivalent immune response and whether there are and which would be the effects of RB51 revaccination on heifers, despite S19 and RB51 being successful vaccine strains worldwide used in the control of brucellosis. Our efforts were concentrated in an extensive evaluation of the acquired immune response induced after brucellosis vaccination, in order of to understand which mechanisms are involved in the long lasting immune response induced by the classical *B*. *abortus* vaccines. The present study addressed some of these questions and showed that prime-vaccination of calves with S19 or RB51 as well as RB51 revaccination induce a strong and complex immune response dominated by Th1 profile, although after RB51 revaccination the differences between immune profiles induced by prime-vaccination become more accentuated.

Our results showed that vaccination with S19 or RB51 and RB51 revaccination induce a significant blastogenic response of both major T lymphocytes subpopulation, CD4^+^ and CD8^+^, indicating that both subsets are involved in the protection conferred by these *B*. *abortus* vaccines in cattle (Fig 3). Indeed, the resistance to *B*. *abortus* infection in mice has been credited to coordinated action of CD4^+^ and CD8^+^ T-cells [61–64]. After brucellosis vaccination in cattle, CD4^+^ T-cells have been implicated as the main source of IFN-ɣ, whereas CD8^+^ T-cells which were proliferating differentiate into cytotoxic effectors cells (Figs 4 and 5) [41]. However, a different polarization of the immune response, CD4^+^- or CD8^+^-dominant, was observed after the booster with RB51, for S19 and RB51 prime-vaccinated animals, respectively. These results suggest that the vaccine strain used in the calfhood immunization directs the profile of the immune response observed after RB51 booster performed on heifers, which is CD4-directed in S19 prime-vaccinated animals and CD8-directed in RB51 prime-vaccinated animals. This CD8-dominant blastogenic response following RB51 revaccination in RB51 group is supported, considering that the RB51 prime-vaccinated animals also showed a significant higher expression of both perforin and granzyme B by CD8^+^ T-cells compared to the S19 group. Perforin, a pore-forming protein, and granzyme B, a serine protease, are upregulated and synergistically involved in the lytic activity triggered chiefly by CD8^+^ T-cells after CD3 / TCR activation [65,66]. Beyond RB51 booster, vaccination with S19 or RB51 also elicited a significant up regulation in expression of granzyme B on CD8^+^ T-cells, while expression of perforin was significantly increased only in RB51 group. These results indicate that both vaccines induce specific cytotoxic activity exercised by CD8^+^ T-cells, however; it appears to be slightly stronger following RB51 vaccination. Similarly to the present findings, it was also previously demonstrated in mice that RB51 vaccination induced specific cytotoxic activity, mainly by CD8^+^ T lymphocytes [19]. Furthermore, studies in gene-disrupted mice also showed that MHC I dependent CD8^+^ T-cells has a great impact on the acquisition of resistance to infection by *B*. *abortus* [67]. Nevertheless, to the best of our knowledge, this is the first report describing the role of CD8^+^ T-cells in the immune response induced in cattle by brucellosis vaccination employing S19 and RB51. Our findings, as well as previous results using mouse model, indicate that the protective immune response induced by vaccination with S19 or RB51, and by RB51 revaccination is characterized primarily by synergistic activity of CD8^+^ cytotoxic T-cells and IFN-ɣ-producing CD4^+^ T-cells.

CD4^+^ T-cells are definitely the main source of IFN-ɣ following brucellosis vaccination in cattle. Data from the present study on intracellular expression of IFN-ɣ by CD4^+^ and CD8^+^ T-cells confirm our previous report [41]. Besides, IFN-ɣ, CD4^+^ T-cells also demonstrated to be the main source of IL-17A, a key cytokine in the development of a Th17 immune response, which has been implicated in autoimmune and autoinflammatory diseases, but also has proven to be significant in overcoming several infectious diseases [68]. The pattern of expression of IFN-ɣ and IL-17A by CD4^+^ T-cells was similar between both vaccination regimens until day 365, in which the peak of expression was observed (Fig 5). We speculate that this apparent higher expression of IFN-ɣ and IL-17A by CD4^+^ T-cells on day 365, in fact reflects the increased number of IFN-ɣ- or IL17A-expressing CD4^+^ T-cells due to clonal expansion of memory cells, rather than the amount of cytokine produced by those cells. This hypothesis is widely supported taking into account that the IFN-ɣ accumulated in the cell culture supernatants measured by ELISA did not show this increased production on day 365 (Fig 6). Additionally, the evaluation of the mean of fluorescence intensity of IFN-ɣ or IL-17A on CD4 T-cells also showed lower values at day 365 compared to the other time points assessed (data not shown).

In contrast to the similar IFN-ɣ profile on T-cells post prime-vaccination, following revaccination, only the group vaccinated with RB51 twice did not decrease the IFN-ɣ levels, which was significantly higher compared to S19 group on both CD4^+^ and CD8^+^ T-cells. Similarly, the response of CD4^+^IL-17A^+^ T-cells was significantly higher in RB51 revaccinated animals compared to S19 group (day 393) (Fig 5). In addition, the results of memory markers on CD4^+^ and CD8^+^ T-cells, after revaccination with RB51, also exhibited a significant increase in RB51 group (day 365 vs. 393) (Fig 7). These differences in the immune profile between the vaccination regimens observed post-revaccination could be attributed to the dose of vaccine used or to individual aspects of both brucellosis vaccines tested. Since the dose of S19 (0.6–1.2 x 10^11^ CFU) used was higher than the dose of RB51 (1.3 x 10^10^ CFU) [48,49], it is tempting to speculate that the significant increase in CD4^+^IFN-ɣ^+^ and CD8^+^IFN-ɣ^+^ response observed in animals vaccinated twice with RB51 compared with S19 group, may have occurred due the lower dose of RB51 used in both vaccinations. This also could explain the absence of significant increase of CD8^+^CD45RO^+^ response in S19 group following the RB51 revaccination. It seems that, as result of the larger dose of S19 used compared to RB51, after prime-vaccination there was a high stimulation of the immune system in S19 group that could not be enhanced by the RB51 revaccination, different from that observed for the RB51 group. This impression is supported considering that RB51 is more attenuated than S19, as several studies have demonstrated that the clearance of S19 is longer than RB51 in spleen of infected mice and in lymph nodes of cattle after immunization [12,69], besides causing severe placentitis and fetal death in pregnant mice [70]. Moreover, analysis of the IFN-ɣ accumulated in the cell supernatant culture confirming the longer persistence of immune stimulation given by vaccination with S19, as only S19 prime-vaccinated animals exhibited significant production of IFN-ɣ on day 210 compared to day 0 (Fig 6). Likewise, data on the evaluation of the mean of fluorescence intensity of MHC class II on CD4^+^ T-cells also showed significant increase only to S19 group in comparison of day 0 with day 210 (S1 Fig). The expression of MHC class II on T-cells is an important marker of activation of these cells, besides being functional, as it can present peptide antigens to other T-cells [71]. Furthermore, compared to day 0, a significant higher expression of memory marker by CD4^+^ and CD8^+^ T-cells was observed on day 210 only in S19 group (Fig 7), suggesting that S19, but not RB51 vaccination induced long-lived CD4^+^ memory cells.

However, it is noteworthy that although we have observed a greater persistence of immune stimulation in animals vaccinated with S19, evidenced by prolonged IFN-ɣ, MHC Class II^+^CD4^+^ cells and CD4^+^ memory cells response, both vaccination regimens were able to evoke a significant IFN-ɣ response after vaccination and revaccination (Fig 6). Corroborating these findings, Singh et al. [40] also observed that RB51 vaccinated cattle have an IFN-ɣ response in the peripheral blood up to 60 days after vaccination, which was not detected at 90 days post-vaccination. Moreover, the significant induction of CD4^+^IFN-ɣ^+^ T-cells after S19 or RB51 vaccination and RB51 revaccination (RB51 group), as well as the absence of an IL-4 response, characterize the development of a predominant Th1 immune response following brucellosis vaccination in cattle. The central role of IFN-ɣ in the protection against brucellosis is recognized once IFN-ɣ knockout mice died due to brucellosis and IFN-ɣ deficiency is more severe than CD8^+^ T-cells or IL-12 deficiency to overcome the infection in mice [72,73]. Besides Th1 immune response, our results also showed that Th17 subset cells were significantly stimulated by S19 and RB51 vaccination (Fig 5). Th17 cells appears to act synergistically with Th1 cells, suggesting that they may have a protective role in oral RB51 and recombinant unlipidated Omp19 vaccination of mice, mainly by mucosal immunity [20,74]. Despite protection has not been assessed, the induction of Th1 and Th17 cell subsets observed after brucellosis vaccination in cattle suggests that these cells are involved in the protective immunity conferred by vaccination (Fig 9).

CD4^+^ and CD8^+^ memory cells were also elicited by S19 and RB51 vaccination, although only S19 vaccination stimulated the development of CD21^+^ memory cells. Memory cells are a critical parameter to be assessed in the long-term immune response to a vaccine, as *B*. *abortus* vaccines. The desirable long-term protection requires generation of immune memory cells capable of rapid and effectively reactivation upon subsequent microbial exposure [75]. Therefore, the increase in CD4^+^ and CD8^+^ memory cells following S19 and RB51 vaccination and RB51 revaccination (RB51 group) suggest that this may be one of the mechanism used by these classical *B*. *abortus* vaccines to induce protection in cattle, as Tc1 CD8^+^ and Th1 CD4^+^ T-cells are major immune defenses against *B*. *abortus* [76]. Differently, only S19 vaccinated animals induced B memory cells post-vaccination, which could be explained taking into account the differences in LPS composition between S19 and RB51. Lipopolysaccharide O-side chain is an immunodominant antigen of *B*. *abortus*, to which the majority of antibodies resulting from immunization or natural infection are directed, being expressed in S19 and absent in RB51 [15,77]. This highly deficient expression of the LPS O-side chain by RB51 is probably also the explanation to the markedly lower antibody production after the first vaccination in RB51 group compared to animals vaccinated with S19 (Fig 8). In fact, when animal sera were tested against antigens from the cell-lysed vaccine strains the difference between the two vaccines after vaccination was markedly reduced (S2 Fig). Interestingly, the antibody profile observed in both vaccination regimens was similar following vaccination and revaccination and it was predominantly IgG1. This result was in contrast to the profile observed in cellular immune response assessed, which was predominantly Th1, given that in cattle as well as human and mouse IgG1 isotype appears to be associated to a Th2 response, whereas IgG2 isotype is more related to a Th1 response [78]. The almost opposite findings observed to cellular and humoral immune response after brucellosis vaccination and revaccination should be understood considering that the exact contribution of humoral immunity in resistance to *B*. *abortus* infection is not well established, while the response mediated by cells have been proven to be crucial to overcome the infection [62,63]. Moreover, it is not known whether the Th1 / Th2 polarization very well observed in mouse also occurs in the same way in cattle. Furthermore, the intricate interaction between the host and the pathogen usually demands a balance between Th1 and Th2 response.

Also between proinflammatory and anti-inflammatory cytokines, a balance is required, so that an optimal immunological response is established. IL-10, an anti-inflammatory cytokine, has been implicated in offset production of Th1 cytokines and in downregulation of macrophage effector functions after *B*. *abortus* infection or RB51 vaccination in mice [18,67,79]. However, our results showed that only S19 vaccinated calves exhibited a significant increase in IL-10 production, which was even statistically superior to RB51 vaccinated group at the same point (Fig 6). We speculate that, as result of the slightly higher immunogenicity of S19, demonstrated by the significant production of proinflammatory cytokines as IFN-ɣ and IL-6 compared to RB51, higher levels of IL-10 are necessary probably to avoid an excessive proinflammatory response. Evidences in the literature showed that the phenotype of bovine regulatory T-cells (Treg), the main source of IL-10, may be different of Treg cells from mice and humans, being WC1^+^ ɣδ-cells instead of αβ^+^CD4^+^FoxP3^+^CD25^+^ [80]. Despite the cell source of this cytokine being not assessed, the overlap of the results of IL-10 accumulated in the cell culture supernatant (Fig 6) and the results of CD4^+^FoxP3^+^CD25^+^ T-cells (S3 Fig) suggests that there was no association between the CD4^+^FoxP3^+^CD25^+^ T-cells and IL-10 production, corroborating the hypothesis that the source of IL-10 in cattle probably is another cell subset. As the FoxP3^+^CD25^+^-expressing CD4^+^ or CD8^+^ T-cells seems to be proliferating and CD25 is an IL-2 receptor, it is possible to infer that these cells may represent activated T-cells. Analysis of TGF-β, another anti-inflammatory cytokine, and IL-10 mRNA showed an increase in gene transcription over the experiment for both vaccination regimens evaluated (S4 Fig). However, IL-10 gene transcription seemed not to be related to protein expression, indicating mRNA processing, since results of IL-10 ELISA and qPCR widely disagreed. On the other hand, as the time required for the detection of mRNA and protein are very different and IL-10 mRNA and protein were both assessed after six days of culture, this could explain the different results observed. For TGF-β, the mRNA levels observed need to be broadly investigated as this cytokine has pleiotropic effects, especially in the regulation of effector and regulatory CD4^+^ T-cell responses, and can be secreted by many cell types [81].

Regarding IL-6, our findings revealed a significant increase in the secretion of this cytokine after both S19 and RB51 vaccination, suggesting that the secretion of IL-6 in response to brucellosis vaccination may assist in the development of a Th1 and Th17 response and consequently favor the elimination of the pathogen. Nonetheless, the level of IL-6 significantly decreased after the RB51 revaccination in both vaccination regimens, despite there was an increase in the levels of IFN-ɣ. As IL-6 is proinflammatory cytokine that plays a pivotal role during the transition from innate to acquired immunity, it is possible to infer that the reduction in IL-6 observed after RB51 revaccination may be a reflection of the higher number of memory cells instead of naïve cells at the moment of revaccination [82].

The present data showed that RB51 revaccination promote an increase in the immune response regardless if the primary vaccination was performed with S19 or RB51, with some of the parameters assessed being even higher in animals prime-vaccinated with RB51 compared to animals prime-vaccinated with S19 (Fig 9). These results strengthen the argument in favor of use of RB51 revaccination in regions where brucellosis is present. However, more studies are necessary to determine which should be the minimum or better interval between the vaccinations and how many vaccinations can or should be performed.

Overall, the present results showed that in cattle the immune response to S19 or RB51 vaccination is characterized by proliferation of specific CD4^+^ and CD8^+^ T-cells; IFN-ɣ and IL-17A production, mainly by CD4^+^ T-cells; cytotoxic activity by CD8^+^ T-cells; IL-6 secretion; induction of CD4^+^ and CD8^+^ memory cells; production of antibodies, mainly of IgG1 isotype; and expression of phenotypes of activation in T-cells. The main differences in the immune response stimulated by S19 compared to RB51 were the higher persistency of the IFN-ɣ response and CD4^+^ memory cells, induction of CD21^+^ memory cells and higher secretion of IL-6 and IL-10. After RB51 revaccination, the immune response was chiefly characterized by increase in IFN-ɣ expression, proliferation of antigen-specific CD4^+^ and CD8^+^ T-cells, cytotoxic CD8^+^ T-cells, higher IL-10 secretion by S19 compared to RB51 group and decrease in IL-6 production in both groups (Fig 9). However, a different polarization of the immune response, CD4^+^- or CD8^+^-dominant, was observed after the booster with RB51, for S19 and RB51 prime-vaccinated animals, respectively. Compared to S19 group after the RB51 booster, RB51 prime-vaccinated animals exhibited significantly higher proliferation of CD8^+^ T-cells, cytotoxic phenotype on CD8^+^ T-cells, expression of IFN-ɣ by CD4^+^ and CD8^+^ T-cells and expression of IL-17A by CD4^+^ T-cells. Globally, the present findings showed that the search for new *B*. *abortus* vaccines should focus not only in IFN-ɣ induction, as it is usually performed. Good candidate vaccines should be able to induce a complex immune response mainly characterized by proinflammatory pattern, cytotoxic CD8^+^ T-cells and Th1 and Th17 CD4^+^ T-cells, but also by some anti-inflammatory profile (IL-10), as demonstrated by the proved brucellosis vaccines. Our results indicate that after first vaccination both vaccine strains (S19 and RB51) induce a strong and complex immune response dominated by Th1 profile, although after RB51 revaccination the differences between immune profiles induced by prime-vaccination become more accentuated (Fig 9).

## Supporting Information

S1 Fig Mean of fluorescence intensity of MHC class II on CD4^+^ T-cells of S19 and RB51 prime vaccinated, and RB51 revaccinated cattle upon in vitro stimulation with ɣ-irradiated *B*. *abortus* 2308. (TIF)

S2 Fig Antibody profile of S19 and RB51 prime vaccinated, and RB51 revaccinated cattle measured by I-ELISA using S19 and RB51 lysed heat-killed antigens (TIF)

S3 Fig Expression of FoxP3 and CD25 by CD4^+^ and CD8^+^ T-cells of S19 and RB51 prime vaccinated, and RB51 revaccinated cattle upon in vitro stimulation with ɣ-irradiated *B*. *abortus* 2308. (TIF)

S4 Fig mRNA level of IL-10 and TGF-β in peripheral blood mononuclear cells of S19 and RB51 prime vaccinated, and RB51 revaccinated cattle upon in vitro stimulation with ɣ-irradiated *B*. *abortus* 2308. (TIF)

S5 Fig Standard tube agglutination test (STAT) and 2-mercaptoethanol test (2ME) of S19 and RB51 prime vaccinated, and RB51 revaccinated cattle. (TIF)
