# Supplementary figures and images for: Immune Response of Calves Vaccinated with Brucella abortus S19 or RB51 and Revaccinated with RB51
Source: PLoS One. 2015 Sep 9;10(9):e0136696. doi: 10.1371/journal.pone.0136696 (PMC4564183; doi:10.1371/journal.pone.0136696)

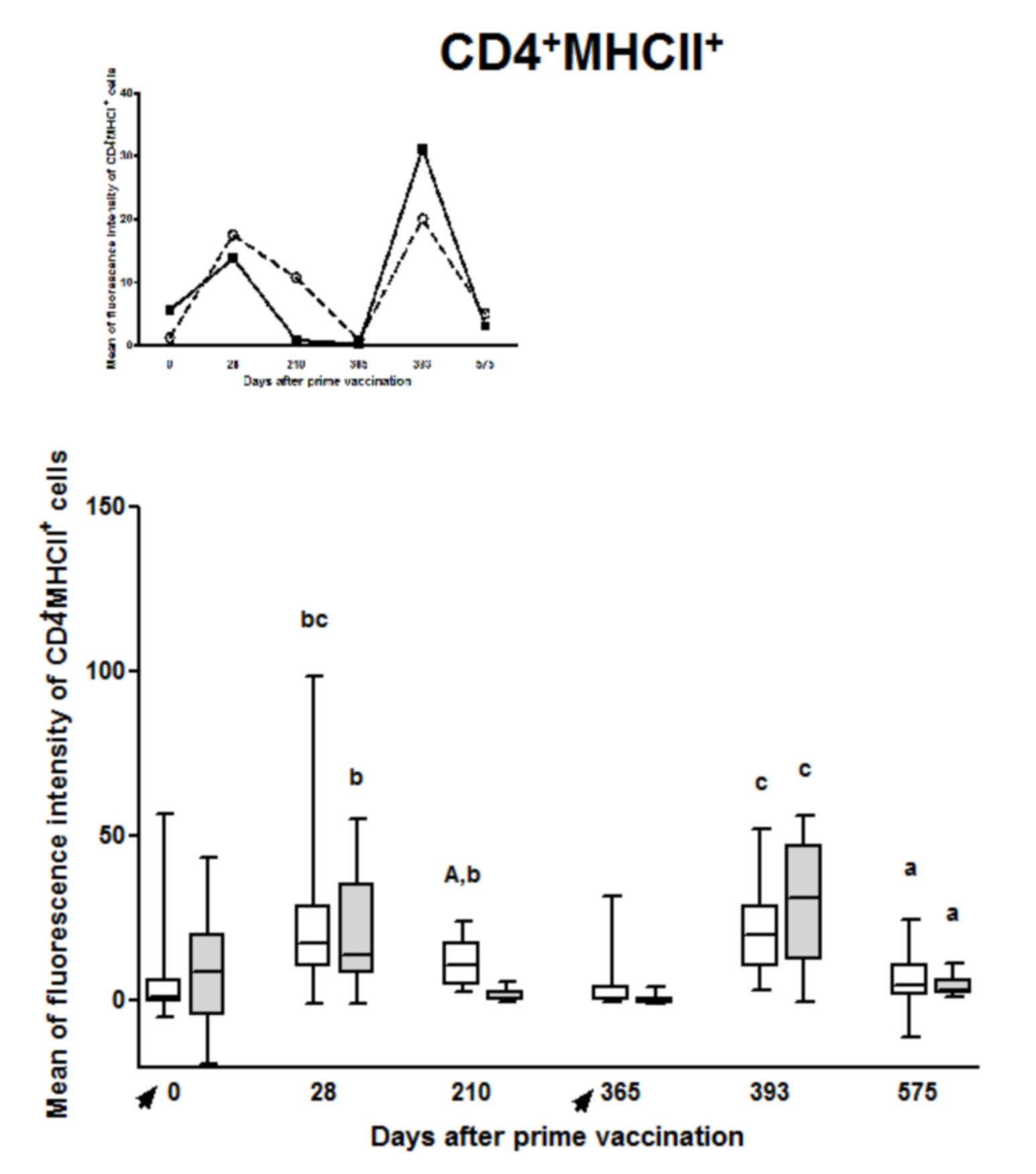

Supplement: S1 Fig — (TIF) [file pone.0136696.s001.tif]

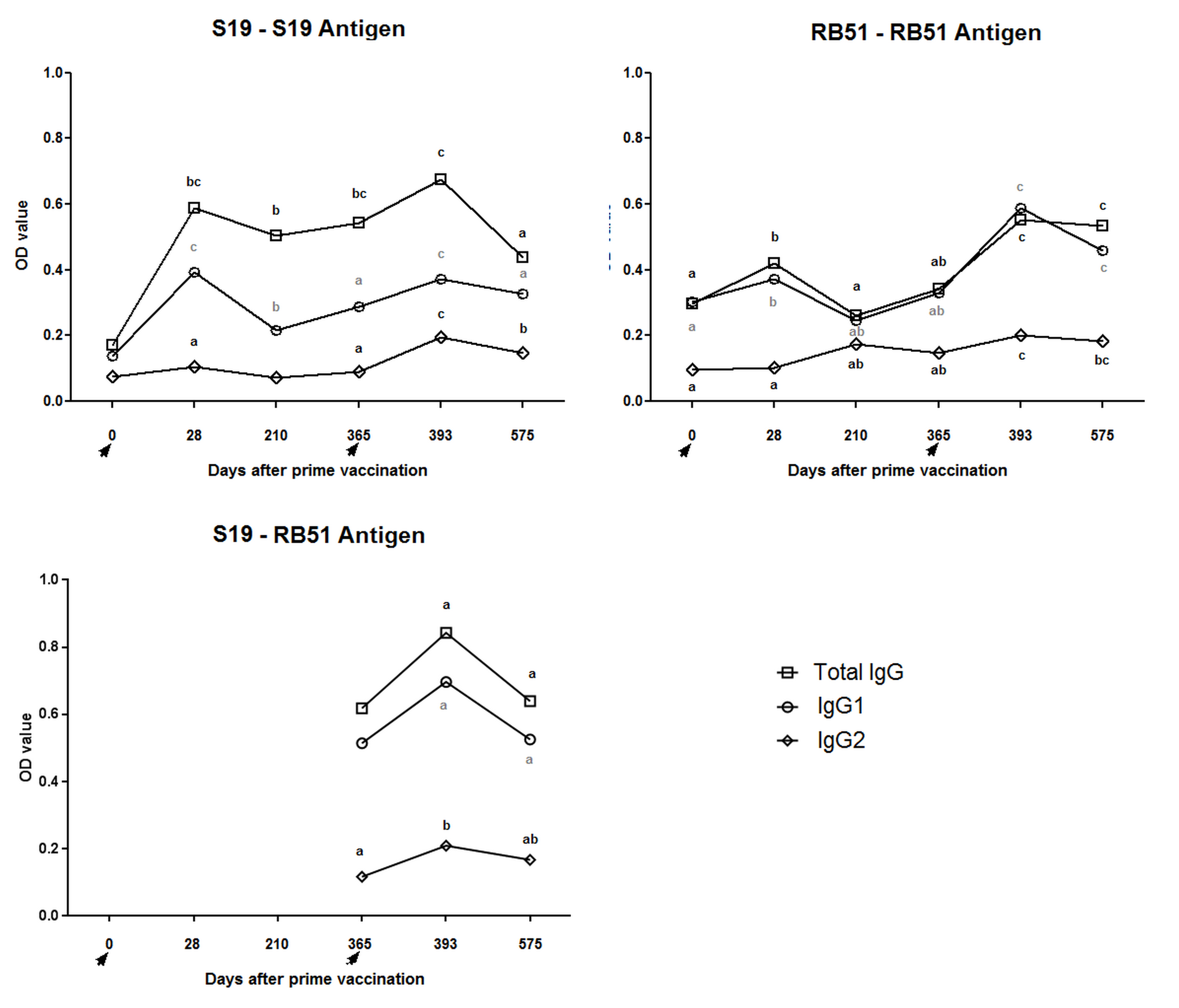

Supplement: S2 Fig — (TIF) [file pone.0136696.s002.tif]

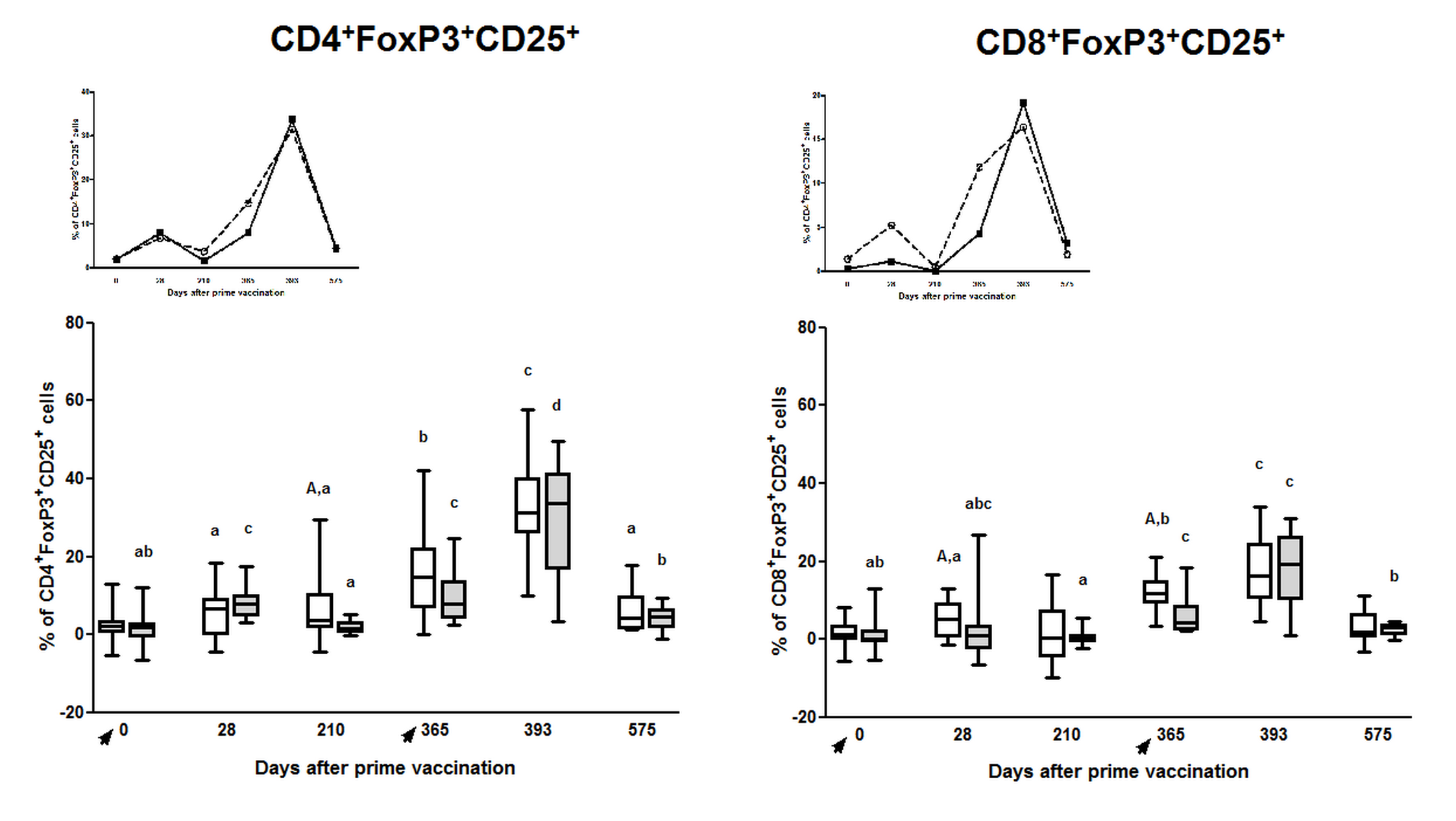

Supplement: S3 Fig — (TIF) [file pone.0136696.s003.tif]

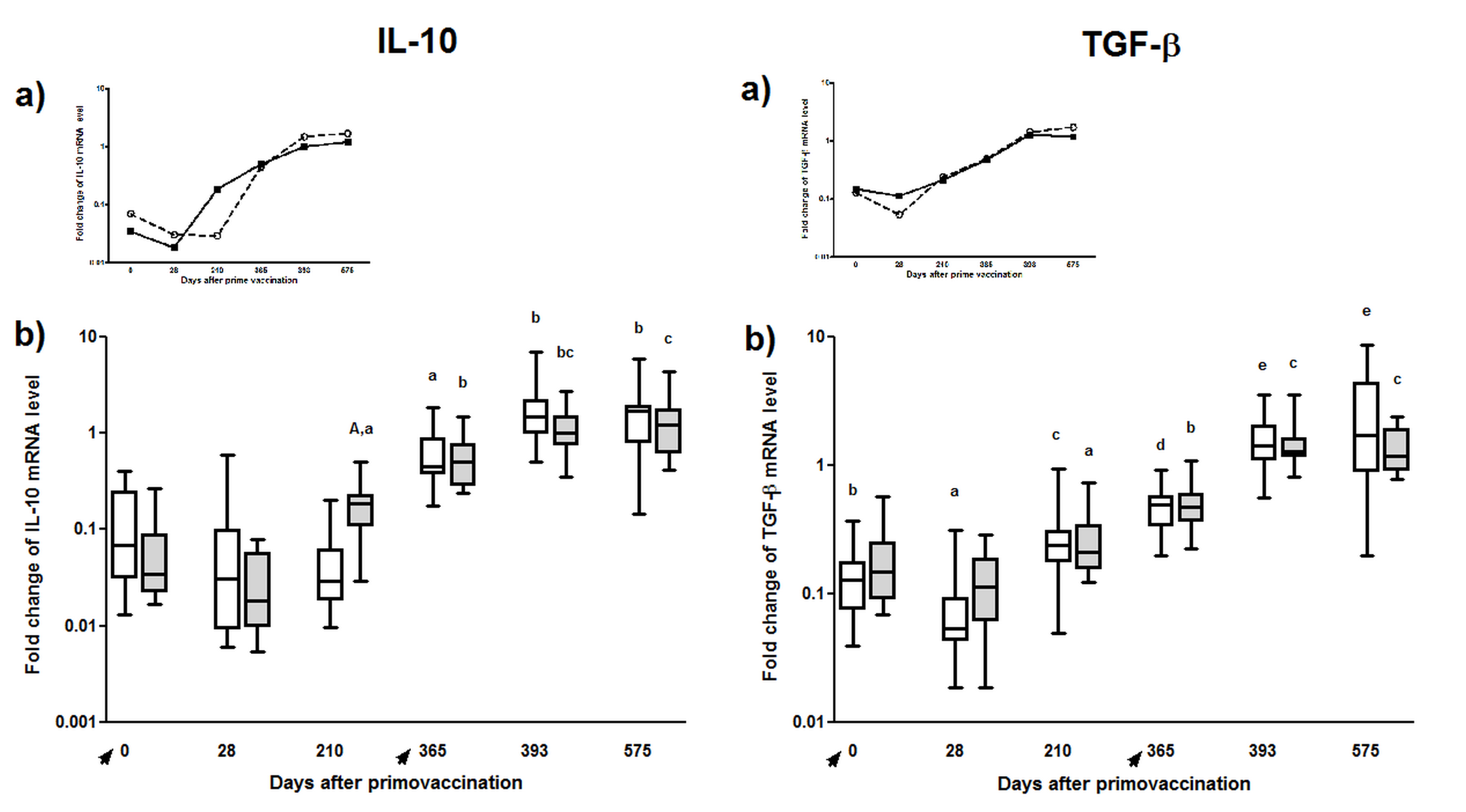

Supplement: S4 Fig — (TIF) [file pone.0136696.s004.tif]

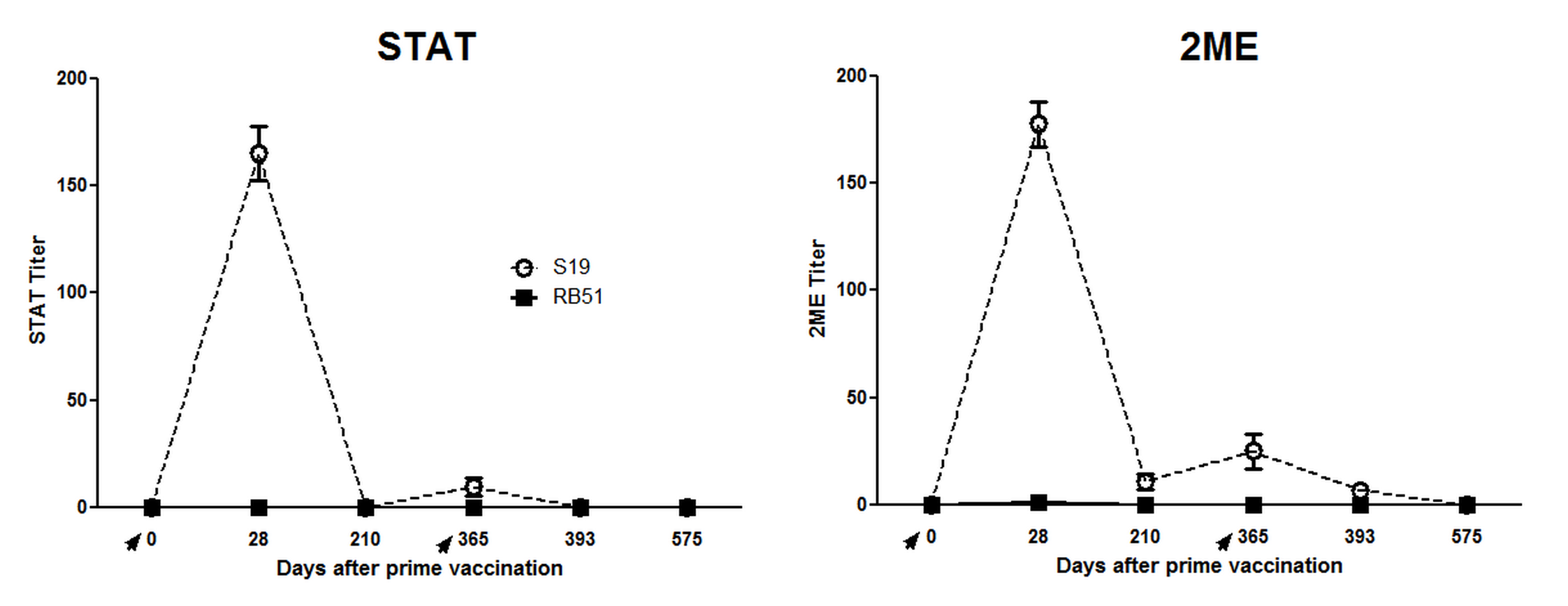

Supplement: S5 Fig — (TIF) [file pone.0136696.s005.tif]
